# Horizontal Gene Transfer and Fusion Spread Carotenogenesis Among Diverse Heterotrophic Protists

**DOI:** 10.1093/gbe/evad029

**Published:** 2023-02-20

**Authors:** Mariana Rius, Joshua S Rest, Gina V Filloramo, Anna M G Novák Vanclová, John M Archibald, Jackie L Collier

**Affiliations:** School of Marine and Atmospheric Sciences, Stony Brook University; Department of Ecology and Evolution, Stony Brook University; Department of Biochemistry and Molecular Biology, Dalhousie University, Halifax, Nova Scotia, Canada; Faculty of Science, Charles University, BIOCEV, Vestec, Czechia; Present address: Institut de Biologie de l'École Normale Supérieure, Paris 75005, France; Department of Biochemistry and Molecular Biology, Dalhousie University, Halifax, Nova Scotia, Canada; School of Marine and Atmospheric Sciences, Stony Brook University

**Keywords:** phytoene synthase, phytoene desaturase, lycopene cyclase, carotenoid oxygenase, phylogenetics, thraustochytrids

## Abstract

Thraustochytrids (phylum: Labyrinthulomycota) are nonphotosynthetic marine protists. Some thraustochytrids have *crtIBY*, a trifunctional fusion gene encoding a protein capable of β-carotene biosynthesis from geranylgeranyl pyrophosphate. Here we show that *crtIBY* is essential in, and encodes the sole pathway for, carotenoid biosynthesis in the thraustochytrid *Aurantiochytrium limacinum* ATCC MYA-1381. We explore the evolutionary origins of CrtIBY and discover that the closest related protein domains are present in a small but diverse group of other heterotrophic protists, including the apusomonad *Thecamonas trahens* and the dinoflagellates *Oxyrrhis marina* and *Noctiluca scintillans*. Each organism within this cluster also contains one or more β-carotene 15-15′ oxygenase genes (*blh* and *rpe65*), suggesting that the acquisition of β-carotene biosynthesis genes may have been related to the production of retinal. Our findings support a novel origin of eukaryotic (apo)carotenoid biosynthesis by horizontal gene transfer from Actinobacteria, Bacteroidetes, and/or Archaea. This reveals a remarkable case of parallel evolution of eukaryotic (apo)carotenogenesis in divergent protistan lineages by repeated gene transfers.

**Significance**

Although most organisms capable of carotenoid biosynthesis are phototrophic, some nonphototrophic eukaryotes have gained, by horizontal gene transfer, the capacity to synthesize carotenoids. This study examines the evolutionary origins of carotenoid biosynthesis proteins in one such group and discovers a set of related proteins in surprisingly diverse eukaryotic lineages including thraustochytrids, dinoflagellates, and apusomonads. In addition to uncovering a novel origin of eukaryotic carotenoid biosynthesis, this research reveals that repeated horizontal gene transfer enabled the parallel evolution of carotenoid biosynthesis in heterotrophic protists.

## Introduction

**Fig. 1.**
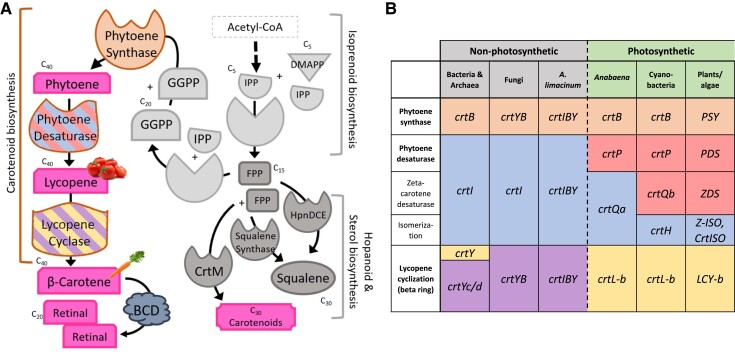
β-carotene biosynthesis is accomplished by orthologous enzymes across taxa. (*A*) Generalized flow-chart of the isoprenoid, sterol, and carotenoid biosynthesis pathways. Enzymatic coloration corresponds to orthologs outlined in (*B*). (*B*) Orthologous carotenoid biosynthesis genes in nonphotosynthetic and photosynthetic organisms (expanded from Alcaíno et al. 2016 and Sandmann 2001). Genes that are orthologous are shaded similarly; fusion genes are indicated by concatenation (e.g., *crtIBY* is a fusion gene of *crtI*, *crtB*, and *crtYc/d*).

Carotenoids are a class of over 1,200 mainly yellow, orange, or red fat-soluble natural isoprenoid pigments characterized by a rigid conjugated hydrocarbon backbone. Key functions of carotenoids are their ability to quench free radicals, thereby acting as antioxidants (Britton 1995; Fiedor et al. 2005), and their role as precursors of apocarotenoids such as retinal, the chromophore for opsin proteins (Spudich et al. 2000). Carotenoids are universally present in photoautotrophs (Hirschberg et al. 1997) and are also found in some nonphotosynthetic bacteria, archaea, and eukaryotes (Britton 1995). Production of β-carotene (C_40_) from two geranylgeranyl pyrophosphate (GGPP; C_20_) molecules minimally requires the activity of three enzymes: phytoene synthase (CrtB), phytoene desaturase (CrtI), and lycopene cyclase (CrtY or CrtYc/CrtYd) (fig. 1*A*). Alternative enzymes for β-carotene synthesis from GGPP, which are specific to organisms with current or past photosynthetic capacity, include several isomerases involved in the conversion from phytoene to lycopene (fig. 1*B*).

Phylogenetic analysis of CrtB, CrtI, and CrtYc/d protein sequences suggests that carotenoid biosynthesis is an ancient process, one that in bacteria has been impacted by extensive horizontal gene transfer (HGT) (Klassen 2010). The origin of carotenoid biosynthesis in many oxygenic phototrophic eukaryotes is associated with endosymbiotic gene transfer during the acquisition of a plastid (Coesel et al. 2008, Frommolt et al. 2008). In contrast, carotenoid biosynthesis in nonphotosynthetic (i.e., heterotrophic) eukaryotes such as the fungi *Xanthophyllomyces dendrorhous* (*Phaffia rhodozyma*) (Andrews and Starr 1976; Verdoes et al. 1999) and *Rhodotorula* spp. (Nakayama et al. 1954) is suggestive of carotenogenesis acquisition via an ancient HGT event (Sandmann 2002). Similarly, arthropod lineages (pea aphids, adelgids, gall midges, spider mites, chiggers, and velvet mites) acquired carotenogenesis via three independent HGT events from fungi (Altincicek et al. 2012, Cobbs et al. 2013, Novakova and Moran 2012).

Among heterotrophic Stramenopila, carotenoid production occurs in the basal-branching and ecologically fungus-like thraustochytrids (phylum: Labyrinthulomycota) (Galasso et al. 2017). Recent work in the thraustochytrid *Aurantiochytrium* sp. strain KH105 revealed a trifunctional carotenogenic fusion gene (*crtIBY*) that by itself confers the ability to produce β-carotene when introduced in yeast (Iwasaka et al. 2018). *crtIBY* is also found in other thraustochytrids, including *Aurantiochytrium* sp. FCC1311 and T66, *Schizochytrium* sp. CCTCC M209059, *Thraustochytrium* sp. ATCC 26185 (Iwasaka et al. 2018), and *Aurantiochytrium limacinum* ATCC MYA-1381 (this study). Neither the selective advantage nor the evolutionary origin of carotenoid biosynthesis in thraustochytrids is clearly understood. Thraustochytrids are the only known heterotrophic carotenogenic stramenopiles, although the distantly related crown group of photosynthetic stramenopiles, the Ochrophyta, also produce carotenoids.

Here we show that inactivation of *crtIBY* in *A. limacinum* ATCC MYA-1381 results in the loss of carotenoid production, revealing that *crtIBY* is the sole carotenogenesis pathway in the organism. We describe the phylogenies of six carotenoid biosynthesis domains, including the three domains in CrtIBY, two β-carotene cleavage genes, and an alternate lycopene cyclase. An unexpected cluster of unrelated nonphotosynthetic eukaryotes was identified in the phylogenies of four of the six protein domains, indicating that (apo)carotenoid biosynthesis in this diverse assemblage represents a dramatic case of parallel evolution by repeated HGT.

## Results

### The *crtIBY* Fusion Gene Encodes the Carotenogenesis Pathway in *A. limacinum*

**Fig. 2.**
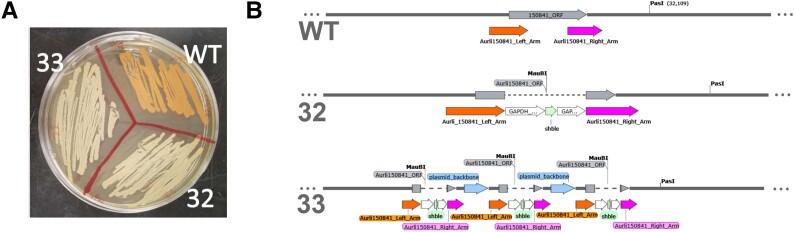
Inactivation of *crtIBY* in *A. limacinum.* (*A*) Agar plate streaks of WT with natural carotenogenic pigmentation relative to the two pigment-less *crtIBY* KOs (32 and 33). (*B*) Annotated genome maps (SnapGene) generated from nanopore sequencing of the *A. limacinum* (WT) and two KOs (32 and 33) reveal an intact *crtIBY* locus in WT and disrupted, yet successful integration of *shble* in the *crtIBY* open reading frame in the two KOs. Sequences indicate a double homologous recombination event having occurred in 32, while a triple tandem repeat integration event occurred in 33. Both integration events resulted in a nonfunctional form of the CrtIBY protein.

Wild-type (WT) *A. limacinum* ATCC MYA-1381 (henceforth *A. limacinum*) colonies produce a marked orange pigmentation when grown in rich media (fig. 2*A*). Using homology searches, we identified the putative trifunctional carotenogenic gene *crtIBY* in the complete genome of *A. limacinum* and targeted it for the genetic knockout by double homologous recombination. We recovered stable zeocin-resistant colonies after electroporation with a construct replacing part of the *crtIBY* coding region with a zeocin resistance gene (*shble*) expression cassette (supplementary fig. S1, Supplementary Material online). Several of these colonies were stark white, in contrast to orange WT colonies, as expected for successful inactivation of carotenoid biosynthesis (streaked colonies 32 and 33 in fig. 2*A*). In addition, these colonies lacked spectrophotometrically detectable carotenoids (supplementary fig. S2*A*, Supplementary Material online) and maintained similar growth rates to WT *A. limacinum* (supplementary fig. S2*B*, Supplementary Material online). In both isolates (32 and 33), inactivation of the *crtIBY* locus had occurred by integration of *shble*, as confirmed by polymerase chain reaction (PCR) (supplementary fig. S3*A*–*C*, Supplementary Material online), Southern blotting (supplementary fig. S3*D*and*E*, Supplementary Material online), and Oxford nanopore long-read DNA sequencing of the complete genome of the knockouts (KOs) (fig. 2*B*). The two KOs differed in the nature of the integration: isolate 32 underwent a simple double homologous recombination replacement event, while colony 33 underwent a triple tandem repeat integration event. Although we did not complement, the presence of multiple colonies with consistent phenotypes and no apparent alternate modifications to the genomes (based on nanopore sequence data) suggests that no other genes are involved in the resulting phenotype. Together, these results confirm that *crtIBY* is necessary for carotenoid biosynthesis in *A. limacinum* and indicate that no alternative carotenoid biosynthesis pathway is present.

**Fig. 3.**
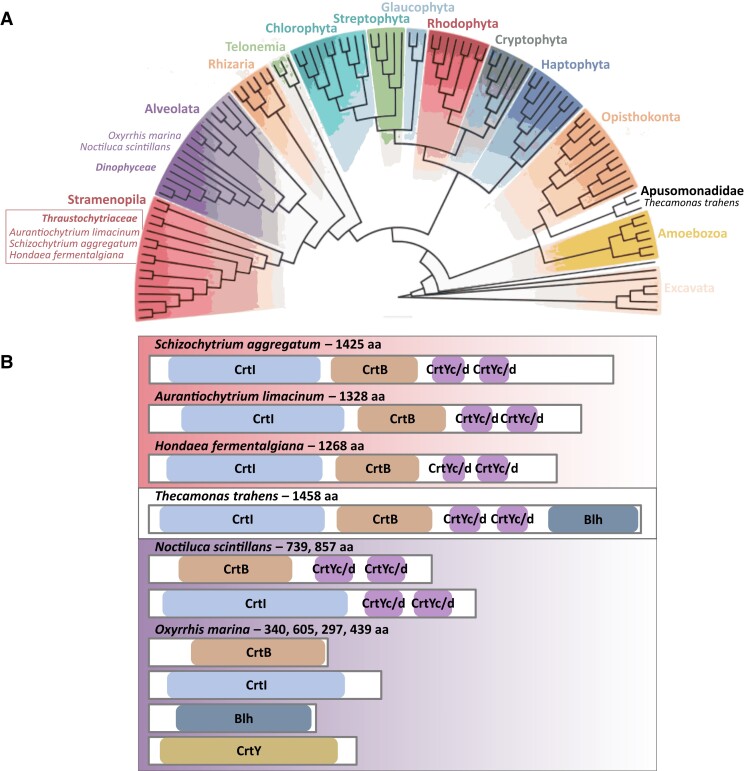
SAHNTO phylogenetic distribution and organization of carotenoid biosynthesis domains. (*A*) Schematic of divergent eukaryotic lineages, modified from Charon et al. (2020). Indicated are all SAHNTO members: the thraustochytrids *A. limacinum, S. aggregatum, H. fermentalgiana* (Stramenopiles, *Thraustochytriaceae*); the dinoflagellates *N. scintillans* and *O. marina* (Alveolates, *Dinophyceae*); and the apusomonad *T. trahens* (Apusomonadidae). (*B*) Protein domain organization diagrams (N- to C-terminus; domain shading is consistent with fig. 1): CrtB—phytoene synthase, CrtI—phytoene desaturase, CrtYc/d—lycopene cyclase, Blh—β-carotene 15-15' oxygenase (BCD superfamily), and CrtY—phototrophic lycopene cyclase. A CrtI, CrtB, CrtYc/d trifunctional multidomain protein is found in the thraustochytrids: *S. aggregatum* (Schag101501), *A. limacinum* (Aurli_150841), *H. fermentalgiana* (re-annotated A0A2R5GF32; see Supplementary Material online); whereas a CrtI, CrtB, CrtYc/d, Blh quadrifunctional multidomain protein is found in *T. trahens* (XP_013761525.1). Two bifunctional multidomain proteins are found in *N. scintillans* as CrtB, CrtYc/d and CrtI, CrtYc/d (CAMPEP0194550082 and CAMPEP0194488352, respectively), whereas in *O. marina* CrtB, CrtI, Blh, and CrtY are found as four single-domain proteins (CAMPEP0205054184, CAMPEP0204966166, CAMPEP0205060456, and CAMPEP0204311066, respectively). Protein lengths in amino acids (aa) are included for reference.

We used basic local alignment search tool (BLAST) to perform a preliminary search of the GenBank nonredundant (nr) database for proteins related to CrtIBY. We only found the CrtIBY multidomain structure in other thraustochytrids and in *Thecamonas trahens*, which belongs to the unrelated eukaryotic lineage Apusomonadidae (fig. 3*A*). *T. trahens* contains a protein with the three carotenoid biosynthesis domains (CrtI, CrtB, CrtYc/d from N- to C-terminus) plus a fourth (C-terminal) domain, which is not found in thraustochytrids (including *A. limacinum*), corresponding to the Blh type of β-carotene 15–15′ oxygenases (BCD) which is involved in the oxidative cleavage of β-carotene to form the apocarotenoid retinal (fig. 1*A*; fig. 3*B*). To further investigate this unexpectedly disjunct taxonomic distribution, we carried out phylogenetic analyses independently on each of the three domains in CrtIBY as well as the Blh domain.

### Four (apo)Carotenoid Biosynthetic Enzymes Reveal an Unexpected Alliance Between Thraustochytrids and Select Other Diverse Eukaryotes

For each of the four (apo)carotenoid biosynthesis domains CrtB, CrtI, CrtYc/d, and Blh, we observed a common phylogenetic pattern: several diverse eukaryotes consistently grouped together. We call this polyphyletic group SAHNTO (*Schizochytrium aggregatum, A. limacinum, Hondaea fermentalgiana*, *Noctiluca scintillans, T. trahens, Oxyrrhis marina*). Each of the carotenoid biosynthesis domains of SAHNTO was sister to homologous domains from lineages of Actinobacteria, Bacteroidetes, or Archaea.

#### Phytoene Synthase in SAHNTO

**Fig. 4.**
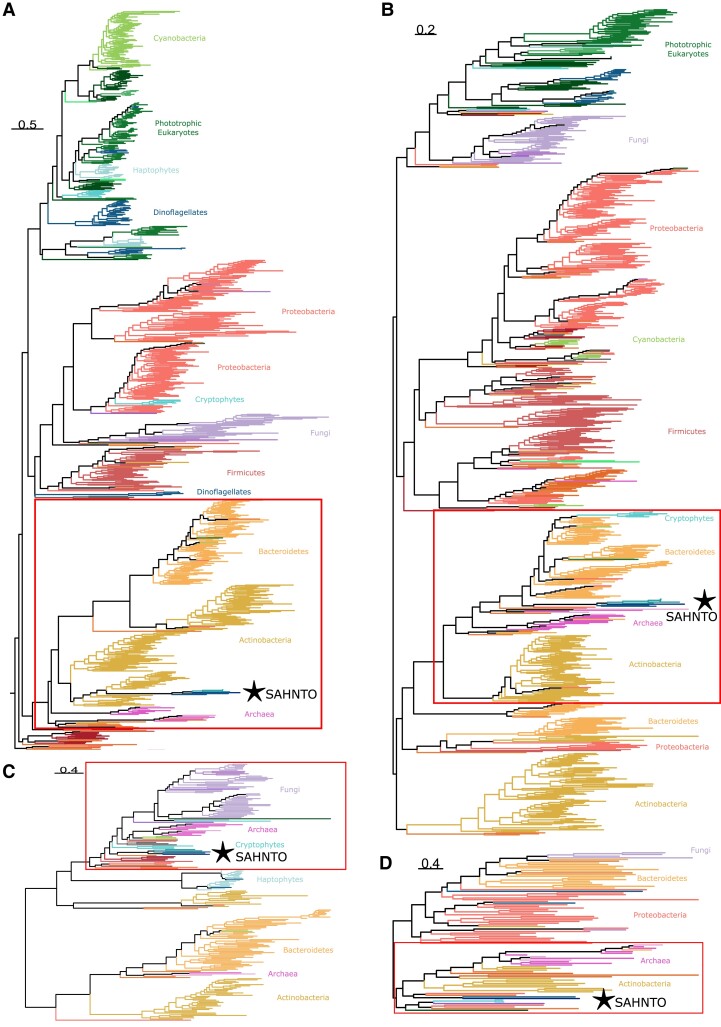
The carotenoid biosynthesis domains from a set of diverse, taxonomically distant eukaryotes called SAHNTO (*S. aggregatum, A. limacinum, H. fermentalgiana, N. scintillans, T. trahens,* and *O. marina*) group together (⋆) in phylogenies of (*A*) CrtB/CrtM/HpnD, (*B*) CrtI/CrtH/Z-ISO/CrtISO, (*C*) CrtYc/d, and (*D*) Blh. For all phylogenies, domain sequences were aligned with multiple alignment using fast fourier transform (MAFFT), retaining positions where less than 90–99% of sequences contained gaps (see Supplementary Material online). ML phylogenies were estimated in IQ-TREE using the best-fit model (supplementary table S5, Supplementary Material online) and midpoint rooted. The CrtB/CrtM/HpnD phylogeny was truncated to remove HpnD sequences. Taxa and node support in the red boxes are magnified in figure 5, and for complete trees see supplementary figures S4–S6 and S8, Supplementary Material online. Scale bars indicate the inferred number of amino acid substitutions per site.

Phytoene synthase (CrtB) catalyzes the first committed step of C_40_ carotenoid biosynthesis: the head-to-head condensation of two GGPP molecules to produce phytoene. The related CrtM and HpnD enzymes catalyze the analogous condensation of two farnesyl pyrophosphate (FPP; C_15_) molecules in the synthesis of C_30_ carotenoids or hopanoid lipids, respectively (fig. 1*A*). We identified four CrtB-containing lineages (as previously reported in Klassen 2010): 1) Proteobacteria and fungi, 2) Firmicutes, 3) Actinobacteria, Bacteroidetes, and Archaea (herein referred to as ABA), and 4) oxygenic phototrophs, including the Ochrophyta (photosynthetic stramenopiles) and Cyanobacteria (fig. 4*A*; supplementary fig. S4, Supplementary Material online). Additionally present were two unexpectedly placed subgroups of photosynthetic eukaryotes including a group of dinoflagellates sister to the Firmicutes and a group of cryptophytes within the Proteobacteria/fungi lineage.

**Fig. 5.**
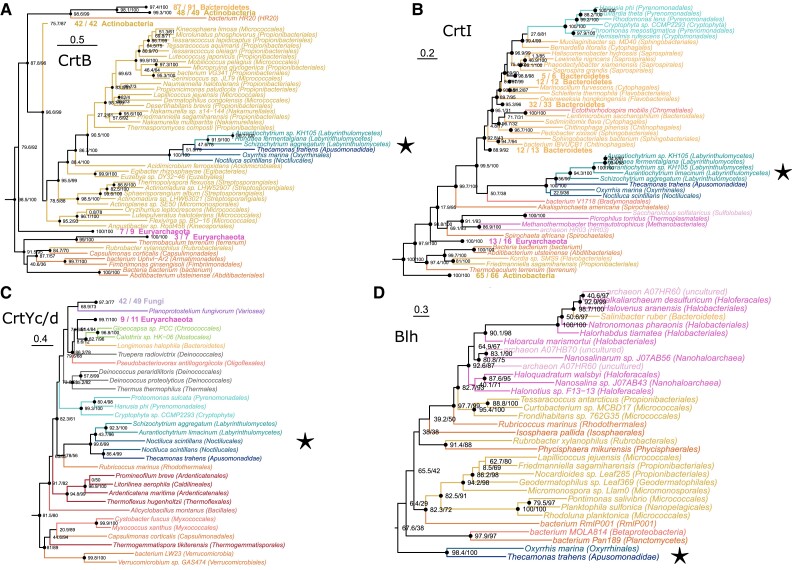
Phylogenetic structure surrounding SAHNTO clusters, including taxa and node support of SAHNTO (⋆) sisters in carotenoid biosynthesis phylogenies: (*A*) CrtB/CrtM/HpnD, (*B*) CrtI/CrtH/Z-ISO/CrtISO, (*C*) CrtYc/d, and (*D*) Blh. Values indicate the results of the SH-aLRT and UF of 1,000 replicates, respectively. For complete trees see supplementary figures S4–S6 and S8, Supplementary Material online. Scale bars indicate the inferred number of amino acid substitutions per site.

Rather than grouping with its closest relatives (photosynthetic Ochrophyta), thraustochytrid CrtB (*S. aggregatum, A. limacinum, H. fermentalgiana*) branched in the SAHNTO cluster with sequences from two nonphotosynthetic dinoflagellates, *O. marina* and *N. scintillans*, and with *T. trahens* (Apusomonadidae) with maximum support [Shimodaira–Hasegawa approximate likelihood ratio test and ultrfast bootstrap approximation (SH-aLRT/UF) 100/100; fig. 5*A*]. Thraustochytrids, dinoflagellates, and apusomonads are taxonomically unrelated to one another (Strassert et al. 2021) and are not united by any other obvious traits. The SAHNTO CrtB group was nested within the ABA CrtB lineage, and specifically within a subset of the Actinobacteria CrtB lineages including representatives from Propionibacteriales, Micrococcales, and Nakamurellales (fig. 5*A*).

Like *A. limacinum,* CrtB in the thraustochytrids *S. aggregatum* and *H. fermentalgiana* was encoded in a trifunctional fusion gene (fig. 3*B*) (see corrected *H. fermentalgiana* gene model in Supplementary material). In contrast, *N. scintillans* CrtB was encoded along with CrtYc/d in a bifunctional fusion gene, while CrtB in *O. marina* appeared to be encoded by a stand-alone gene (fig. 3*B*).

#### Phytoene Desaturase in SAHNTO

Phytoene desaturase (CrtI and related proteins) is involved in the desaturation of phytoene into lycopene (fig. 1*A*). In fungi and nonphotosynthetic bacteria, CrtI also performs isomerization. In phototrophic eukaryotes, homologs Z-ISO and CrtISO isomerize zeta-carotene to form lycopene (Sandmann 2002) (fig. 1*B*). Cyanobacteria have a similar CrtI-related isomerase, CrtH.

Similar to CrtB (phytoene synthase), our phylogenetic analysis of CrtI (fig. 4*B*; supplementary fig. S5, Supplementary Material online) placed most phototrophic eukaryotes (including ochrophytes) in a single group, excluding a cluster of cryptophytes that grouped within the ABA lineage. In contrast to the CrtB phylogeny (although consistent with Klassen 2010), the fungal clade (and metazoans within) branched sister to the photosynthetic eukaryotes rather than grouping with proteobacteria. And conversely, cyanobacteria, rather than grouping with the phototrophic eukaryotes, grouped within the proteobacterial clade.

Strikingly, the CrtI phylogeny again revealed a SAHNTO cluster (100/100) consisting of the same group of diverse species (*S. aggregatum*, *A. limacinum, H. fermentalgiana*, *O. marina*, *N. scintillans,* and *T. trahens*) nested within the ABA lineage. The SAHNTO cluster was sister to a single deltaproteobacterial sequence (suggesting that sequence may be misplaced) and to a group (*n* = 76) of Bacteroidetes, including a large number (*n* = 69) of Chitinophagia, Sphingobacteria, Cytophagia, and Flavobacteria (fig. 5*B*). This group also included a few representatives (*n* = 3) from other bacterial lineages, as well as the aforementioned cryptophyte cluster.

As was the case in *A. limacinum,* CrtI in the thraustochytrids *S. aggregatum* and *H. fermentalgiana* was encoded in a trifunctional fusion gene (fig. 3*B*). In contrast, *N. scintillans* CrtI was encoded in a bifunctional fusion gene with CrtYc/d; this is different from the previously mentioned bifunctional fusion gene encoding CrtB with CrtYc/d. In *O. marina* CrtI appeared to be encoded in a stand-alone gene (fig. 3*B*).

#### Heterodimeric Lycopene Cyclase in SAHNTO Except O. marina

Lycopene cyclase converts the linear lycopene molecule into the first cyclic carotenoid: β-carotene (fig. 1*A*). Independent evolutionary origins of the lycopene cyclase function include CrtY/CrtL, CruP/CruA/CruB, and the heterodimeric CrtYc/d studied here. CrtYc/d is not common in phototrophic eukaryotes (fig. 1*B*), Proteobacteria, or Firmicutes, but was found in many members of the ABA lineage (fig. 4*C*, supplementary fig. S6, Supplementary Material online).

Notably, the phylogeny of CrtYc/d again revealed a SAHNTO-like cluster (99.6/99) containing the thraustochytrids *A. limacinum* and *S. aggregatum* (further analyses revealed *H. fermentalgiana* does contain CrtYc/d, but the domain was not included in our database because of misprediction; see Supplementary material online), *T. trahens,* and two distinct CrtYc/d proteins from *N. scintillans*. However, no copy of CrtYc/d was found in the transcriptomes of *O. marina* (an alternate lycopene cyclase was detected, see below). This modified SAHNTO group is sister to a single Bacteroidetes sequence from the family Rhodothermaceae (fig. 5*C*). This group is sister to a large cluster including another gene from Rhodothermaceae, two cyanobacterial homologs, four Deinococci sequences, and a proteobacterial sequence. In addition, this sister group includes nine halobacterial sequences (Archaea), as well as eukaryotic representatives including four arthropods, two amoebazoan, a green alga, four cryptophytes, and 42 fungal sequences.

Like *A. limacinum,* CrtYc/d in the thraustochytrids *S. aggregatum* and *H. fermentalgiana* was encoded in a trifunctional fusion gene (fig. 3*B*). In contrast, *N. scintillans* had two distinct CrtYc/d-coding genes, each one encoded a bifunctional fusion protein; one also includes CrtB, and the other includes CrtI. CrtYc/d was not detected in *O. marina*, and the relative similarity of the degree of completeness in *N. scintillans* and in *O. marina* transcriptomes (81.2% and 79.5% benchmarking universal single-copy orthologs (BUSCO), respectively; see Supplementary material online) is consistent with the interpretation that *O. marina* lacks *crtYc/d*.

CrtY/CrtL/LCY-b is a lycopene cyclase found primarily in phototrophic eukaryotes (supplementary fig. S7, Supplementary Material online). Some Actinobacteria, Cyanobacteria, and Proteobacteria also contain CrtY, but this lycopene cyclase domain was not detected in Archaea. *O. marina*, the only representative of SAHNTO with CrtY (present as a stand-alone gene, fig. 3*B*), was sister to the branch containing the phototrophs and actinobacterial/cyanobacterial clades.

#### β-carotene Oxygenase in T. Trahens and O. Marina

β-carotene 15-15′ oxygenase (Blh; PF15416) is involved in the oxidative cleavage of β-carotene to produce the apocarotenoid retinal. In our BLAST search, we identified Blh as a fourth (C-terminal) domain in the same *T. trahens* protein containing CrtB, CrtI, and CrtYc/d domains. Blh was also detected in *O. marina* but was not found in any other SAHNTO members (i.e., *A. limacinum, S. aggregatum, H. fermentalgiana,* and *N. scintillans*). In our phylogeny of Blh, a single-domain protein from *O. marina* (fig. 3*B*) was resolved as sister (98.4/100) to the *T. trahens* Blh domain (fig. 4*D*, supplementary fig. S8, Supplementary Material online). The *T. trahens* and *O. marina* Blh domains were sister to a diverse cluster of sequences from Planctomycetes, Proteobacteria, Actinobacteria, and Rhodothermaeota (Bacteroidetes), as well as a group of 12 halobacteria (Archaea) sequences (fig. 5*D*). Outside of this sister group, Blh domains were also present in a cluster of several phototrophic eukaryotes, including cryptophytes and dinoflagellates, as well as fungi, an arthropod, and an amoebozoan.

An alternative β-carotene 15,15′-oxygenase protein family producing retinal from β-carotene is Rpe65 (PF03055). Of all the domains analyzed here, several phyla were found to contain only Rpe65. The Rpe65 phylogeny revealed separate clades of dinoflagellates, fungi, metazoans, and a diverse clade of bacterial and archaeal sequences (supplementary fig. S9, Supplementary Material online). The consistent ABA lineage present in the CrtB and CrtI phylogenies was not observed in the Rpe65 phylogeny.

Five of the six SAHNTO members (all but *T. trahens*) were found to have at least one Rpe65 (all single-domain proteins), but these SAHNTO Rpe65 proteins did not group together in our phylogeny (supplementary fig. S9, Supplementary Material online). The Rpe65 from *N. scintillans* and one of two from *O. marina* were found in the predominantly dinoflagellate clade (both species are dinoflagellates). The thraustochytrids *A. limacinum* and *H. fermentalgiana* each had two Rpe65 proteins and *S. aggregatum* had one. One clade of labyrinthulomycete Rpe65s contained thraustochytrid sequences (including Aurli31778, A0A2R5GWF6, and Schag89143, respectively) and other labyrinthulomycete sequences (*Aplanochytrium stocchinoi* and *Thraustochytrium* sp. LLF1b); this group was sister (98/100) to a cryptophyte (*Geminigera cryophila,* CAMPEP0173101080) and a haptophyte sequence (*Emiliania huxleyi,* CAMPEP0182160074). A second thraustochytrid clade of Rpe65s (including Aurli33046 and A0A2R5G3F8) grouped (95.3/100) with an assortment of eukaryotes, many phototrophic, including dinoflagellates, chlorarachniophytes, and a haptophyte, among others.

## Discussion

We have shown that the polyphyletic SAHNTO group (*S. aggregatum, A. limacinum, H. fermentalgiana*, *N. scintillans, T. trahens,* and *O. marina*) clusters together with strong bootstrap support in independent phylogenies of each of the four (apo)carotenoid biosynthesis domains CrtB, CrtI, CrtYc/d, and Blh. We conclude from this that the four (apo)carotenoid biosynthetic genes (*crtB, crtI, crtYc/d, blh*) were introduced by repeated horizontal gene transfer (HGT) events from the same or similar donor(s) into these diverse lineages. These findings suggest a unique case of repeated HGTs enabling parallel evolution of (apo)carotenoid biosynthesis across the divergent protistan lineages of dinoflagellates, thraustochytrids, and apusomonads. Repeated HGT has been observed previously as a mechanism underlying parallel evolution that resulted in the presence of magnetotaxis across divergent lineages of alphaproteobacteria (Monteil et al. 2020). This study, similarly, reveals parallel evolution of (apo)carotenoid biosynthesis across distant eukaryotic lineages via repeated HGT.

Parallel evolution is when lineages independently evolve a similar genetic change in response to a similar selective pressure (Zhang and Kumar 1997). In the case described here, parallel evolution is indicated by the independent acquisition of the same genetic machinery (CrtIBY and Blh) from the same ancestral condition (lack of (apo)carotenoid biosynthesis) in response to some unknown selective pressure favoring (apo)carotenoid production. The repeated evolution of (apo)carotenoid biosynthesis may also reflect a similar evolutionary solution to more than one selective pressure, made possible by the functional versatility (pleiotropy) of carotenoid production. Carotenoids in the cell can be involved in a variety of functions including oxidative stress response activities (reactive oxygen species [ROS] quenching, free radical scavenging, protection from lipid peroxidation) (Britton 1995) and production of apocarotenoid precursors for rhodopsin-mediated light-dependent activities (phototaxis, transport of ions across membranes) (Spudich et al. 2000). In SAHNTO, the function of carotenoids is not yet clear, although differences in the gene organization of (apo)carotenoid biosynthesis suggest possible differences in function among the different carotenoid-producing SAHNTO taxa. The Rosetta stone hypothesis, which posits that gene fusions may serve as indicators of protein interactions (Marcotte et al. 1999), would imply a functional difference between the quadrifunctional fusion gene of *T. trahens* (*crtI, crtB, crtYc/d, blh*) and the trifunctional fusion gene of thraustochytrids (*crtI, crtB, crtYc/d*): while thraustochytrids may accumulate β-carotene (as the product of CrtIBY), *T. trahens* may cleave β-carotene directly to produce retinal. Consistent with this, numerous thraustochytrids are known to accumulate carotenoids (Valadon 1976; Aki et al. 2003; Carmona et al. 2003; Yamaoka et al. 2004; Armenta et al. 2006; Burja et al. 2006; Fan et al. 2009; Quilodran et al. 2010; Atienza et al. 2012; Gupta et al. 2013; Arafiles et al. 2014; Singh et al. 2015; Zhang et al. 2017; Iwasaka et al. 2018; Park et al. 2018; Jiang et al. 2020; Nham Tran et al. 2020; Leyton et al. 2021), but we could find no evidence of pigment accumulation in *T. trahens* (Droop 1953; Larsen and Patterson 1990; Cavalier-Smith and Chao 2010). With regard to the dinoflagellates, carotenoid compounds in *O. marina* remain unidentified, although concentrated cultures of *O. marina* have a pink pigmentation (Lowe et al. 2011; Jung et al. 2021). Some oceanic *N. scintillans* blooms are red (red tides) (Harrison et al. 2011, and references therein), yet their pigmentation is thought to be from xanthophyll and carotene-rich prey (Balch and Haxo 1984; Shaju et al. 2018; Srichandan et al. 2020). Strains of *N. scintillans* are pale pink in color (Sweeney 1971).

The spread of carotenogenesis by HGT has been inferred previously. The halotolerant marine Bacteroidetes *Salinibacter ruber* acquired carotenoid biosynthesis by HGT from Halobacteria (Mongodin et al. 2005), which are found coinhabiting saltern crystallizer ponds all over the world (Antón et al. 2008). Our phylogenies also support the halobacterial origins of *S. ruber* CrtI and Blh. Another striking example of HGT of carotenoid biosynthesis is observed in arthropods, which acquired carotenogenesis from fungi (Moran and Jarvik 2010; Grbić et al. 2011; Altincicek et al. 2012; Novakova and Moran 2012; Cobbs et al. 2013; Bryon et al. 2017; Dong et al. 2018). This HGT event is also evident in our phylogenies, where several arthropods branch within the fungi in the CrtB, CrtI, and CrtYc/d trees.

SAHNTO (apo)carotenoid biosynthesis domains repeatedly cluster within the ABA group which suggests that ABA was the source of the (apo)carotenoid biosynthesis genes in SAHNTO, revealing a novel origin of carotenoid biosynthesis in eukaryotes. Previously identified eukaryotic phytoene synthases (CrtB) of photosynthetic eukaryotes and fungi are most closely related to cyanobacterial and proteobacterial phytoene synthases, respectively, both in our analyses and in previous studies (Klassen 2010), while SAHNTO CrtBs are related to sequences from the ABA group. Previously identified eukaryotic phytoene desaturases (CrtI) from photosynthetic eukaryotes and fungi are most closely related to cyanobacterial (Frommolt et al. 2008) and proteobacterial phytoene desaturases (Klassen 2010), respectively, while we find SAHNTO CrtI to be most closely related to the ABA group. The phylogenetic affinities of SAHNTO CrtYc/d and Blh domains are less obvious, yet an ABA source is plausible, with sister groups of both SAHNTO CrtYc/d and Blh containing a substantial number of halobacterial (Archaea) proteins.

Despite the macro-phylogenetic consistency of SAHNTO (apo)carotenoid biosynthesis genes grouping together in the ABA lineage, the precise origins of (apo)carotenoid biosynthesis genes in SAHNTO taxa are not obvious. Each of the four SAHNTO carotenogenesis enzymes is most closely related to different taxa in the ABA group: SAHNTO CrtB grouped most closely with Actinobacteria CrtB, while SAHNTO CrtI grouped most closely with Bacteroidetes CrtI, and CrtYc/d and Blh were affiliated with halobacterial genes. Under the hypothesis that a single donor lineage was the source of all four (apo)carotenogenic genetic constituents, their sister group differences may represent a lack of phylogenetic signal, which may result from 1) methodological limitations in reconstructing ancient HGT events involving short signal-poor protein domains; 2) HGT followed by rapid evolution in the new host context, which may further overwrite phylogenetic signal; 3) gene acquisition from an ABA donor lineage that either has not been sampled or is extinct thus representing a ghost of HGT past (Davis et al. 2005); or 4) some combination of the above. Alternatively, a “multiple donors” scenario would entail multiple HGT and fusion events to yield the present-day distribution of carotenoid biosynthesis genes in SAHNTO. We must also consider the likelihood that a single prokaryotic (or viral, see below) donor was itself a recipient of genes acquired via HGT from multiple lineages before the HGTs into eukaryotes. Regardless, the differences in gene organization within SAHNTO taxa (fig. 3*B*) suggest that a combination of gene fusion, fission, and rearrangement events occurred following HGT into the genomes of the different SAHNTO lineages.

It seems unlikely that the acquisition of *crtI, crtB,* and *crtYc/d* in a gene-by-gene fashion would have provided any functional advantage at each step, whereas inheritance of a fusion gene coding for the complete biosynthetic pathway (GGPP to β-carotene or, with *blh*, retinal) immediately yields an antioxidative metabolite and/or opsin-chromophore. In fact, bacterial (apo)carotenoid biosynthesis genes often exist in an operon, suggesting they could have been transferred as a single unit. For example, several Actinobacteria, including *Mycobacterium* spp. (supplementary fig. S10, Supplementary Material online)*, Mycolicibacterium* spp. (supplementary fig. S11, Supplementary Material online), and *Nocardia* spp. (among others; supplementary fig. S12, Supplementary Material online) exhibit a *crtI, crtB, crtYc/d* operon structure. Several Halobacteria (e.g., *Haloarcula* spp.; supplementary fig. S13, Supplementary Material online) have an operon of *crtB, crtYc/d,* and *blh,* with *crtI* elsewhere in the genome. Horizontal operon transfer, enabling all genes in a pathway to be transferred from a prokaryotic donor to a eukaryotic host in a single event, has been observed between an *Escherichia coli* relative (donor) and budding yeasts (Lindsey and Newton 2019). The process of “eukaryotification” of the transferred operon may require the evolution of fewer eukaryotic promoters, as well as simplify the evolution of transcriptional co-regulation and colocalization of protein products, if gene fusion reduces the number of transcription units (Lindsey and Newton 2019). Gene fusion following HGT has been seen previously in bacteria and fungi (Nikolaidis et al. 2014) and in plants (Yang et al. 2016).

SAHNTO species ecology suggests a possible mechanism of HGT for (apo)carotenoid biosynthesis genes. *N. scintillans, O. marina,* and *T. trahens* are phagotrophic (Droop 1953; Larsen and Patterson 1990; Harrison et al. 2011) and bacterivory has also been reported in thraustochytrids (Raghukumar 1992), providing a mechanism to acquire exogenous DNA. Kleptoplasts have been identified in *Noctiluca* (Waller and Kořený 2017, and references therein), supporting the possibility of gene acquisition via phagotrophy. All SAHNTO species are found in tropical and coastal waters (Booth and Miller 1969; Larsen and Patterson 1990; Honda et al. 1998; Raghukumar 2002; Harrison et al. 2011; Watts et al. 2011; Dellero et al. 2018), indicating that the donor lineage(s) likely inhabited the same environment(s).

Alternatively, the circulation and integration of (apo)carotenoid biosynthesis genes may have been facilitated by giant viruses. Two *Mimiviridae* giant viruses (nucleocytoplasmic large dsDNA viruses) that infect choanoflagellates (ChoanoV1 and ChoanoV2) have β-carotene 15-15′ oxygenase (*blh*), phytoene synthase (*crtB*), lycopene cyclase (*crtY*; PF05834; not *crtYc/d*), and phytoene desaturase (*crtI*), adjacent to one another (Needham et al. 2019). Phylogenetic analyses of the ChoanoVirus (apo)carotenoid biosynthetic pathway indicated possible prokaryotic origins and different sister groups for each gene (Needham et al. 2019). These ChoanoViruses also carry genes for three type-I rhodopsins (photoreceptive membrane proteins), suggesting that rhodopsin-based photoheterotrophy may play an important role in host–virus interactions (Needham et al. 2019). It is possible that an undiscovered virus with a similar gene cluster may have been involved in the distribution of the *crtIBY* and *blh* genes in SAHNTO.

Further investigation into the possible link between the acquisition of opsin proteins and *crtIBY* and *blh* genes (either facilitated by the viral opsin-chromophore [rhodopsin-retinal] or otherwise) is warranted. All SAHNTO species possess putative opsin apoproteins. For example, the *O. marina* genome contains over 40 rhodopsin genes; Slamovits et al. (2011) concluded that dinoflagellates acquired proteorhodopsins through at least two independent HGT events from bacteria because one *O. marina* opsin clade groups exclusively with halobacteria and cryptophytes whereas an additional opsin clade groups with fungi. The clustering of *O. marina,* halobacteria, and cryptophyte type-I rhodopsins has been seen in other phylogenies (e.g., Pinhassi et al. 2016) and includes *T. trahens* rhodopsins. This suggests that *O. marina, T. trahens,* cryptophytes, and halobacteria share related rhodopsins, which could also be associated with the transfer of the (apo)carotenoid biosynthetic pathway. McCarren and Delong (2007) found approximately one-third of proteorhodopsin-containing environmental genomic fragments also contain a linked set of retinal biosynthesis genes (*crtB, crtI, crtYc/d, blh*). More work is needed to infer the evolutionary history of rhodopsins in relation to carotenoid biosynthesis.

Our ability to distinguish between different HGT scenarios for the evolution of (apo)carotenogenesis in SAHNTO is presently limited by uncertainty surrounding the timing of the events, large and variable divergence times, and various curious observations gleaned from the phylogenies. For instance, several cryptophytes possess both an ABA-derived and phototroph-related CrtI and contain apparently redundant lycopene cyclases (both CrtYc/d and CrtY) and redundant β-carotene 15-15′ oxygenases (both Blh and Rpe65), suggesting a role for secondary plastid endosymbiosis in the spread of carotenoid biosynthesis within eukaryotic evolution. It is possible that genome-wide scans of the thraustochytrids for HGT will provide insight into the frequency and potential donors of HGT in their evolution. Finally, although unlikely, it is conceivable that the common ancestor of extant eukaryotes had a *crtB, crtI, crtYc/d, blh* biosynthetic gene cluster that underwent independent loss in a massive number of lineages, as well as gene-order rearrangements, duplications, losses, and replacements in other lineages.

## Conclusions

We have shown that experimental disruption of *crtIBY* in *A. limacinum* ATCC MYA-1381 results in a carotenoid-less phenotype. These *crtIBY* knockout, noncarotenogenic *A. limacinum* strains provide an important foundation upon which to investigate the physiological function and contribution of carotenoid biosynthesis to heterotrophic eukaryotic cells. Our phylogenies of CrtIBY domains and Blh (β-carotene 15-15′ oxygenase) reveal a taxonomically diverse cluster (SAHNTO) that includes the thraustochytrids *A. limacinum, S. aggregatum, H. fermentalgiana,* the dinoflagellates *O. marina* and *N. scintillans,* and the apusomonad *T. trahens* (the SAHNTO taxa). The sources of the (apo)carotenogenic genetic constituents are from within ABA lineages, although the four domains each have different affinities. Consistently strong support of a taxonomically disjunct SAHNTO cluster in CrtIBY and Blh phylogenies strongly suggests a case of parallel evolution of (apo)carotenogenesis by repeated HGT from a similar or the same donor. Yet this phenotype homogenization may reflect the ability of the same solution to address different selective pressures: to accumulate carotenoids and/or produce an endogenous supply of retinal. Our results underscore the fact that HGT is a source of phenotypic and biochemical novelty in eukaryotes and that repeated HGT across divergent eukaryotic lineages enabled parallel evolution of (apo)carotenoid biosynthesis in heterotrophic protists.

## Materials and Methods

### *crtIBY* Inactivation

#### Cell Culture Strain

*Aurantiochytrium limacinum* ATCC MYA-1381, deposited by T. Nakahara, was obtained from ATCC. This strain was originally isolated from seawater in a mangrove area of Colonia, Yap Islands, Micronesia (https://www.atcc.org/products/all/MYA-1381.aspx).

#### Plasmid Construction

Primers optimized for In-fusion HD Cloning Plus (Clontech) (supplementary table S1, Supplementary Material online) were used to amplify two 2 kb *crtIBY* (protein identifier [PID]: 150841; 1329 amino acids, referred to as Aurli_150841 hereafter) arms of homology (supplementary fig. S1*A*, Supplementary Material online) from *A. limacinum* genomic DNA. In-fusion cloning was performed twice successively to flank an *A. limacinum-*specific zeocin resistance cassette in the pUC19_GZG backbone (Addgene Plasmid 117226) (Faktorová et al. 2020) resulting in the Aurli_150841_GZG inactivation plasmid (Addgene Plasmid 162563) (supplementary fig. S1*B*, Supplementary Material online).

#### Electroporation and Transformant Stability

*Aurantiochytrium limacinum* ATCC MYA-1381 cells were precultured overnight in 5 ml glucose peptone yeast extract (GPY) media (supplementary table S2, Supplementary Material online) in a 15 ml glass tube, subsequently inoculated into a 250 ml flask of 15 ml GPY media, and grown at 28 °C, 170 rpm for 48 h. Cell preparation, electroporation, outgrowth, and plating were performed as outlined on protocols.io (dx.doi.org/10.17504/protocols.io.qjcduiw). We used AvrII (New England Biolabs [NEB]) to digest Aurli_150841_GZG, which was then column purified (QIAquick PCR Purification Kit, QIAGEN). Electroporation of 1 × 10^8^ cells with 10 μg of cut plasmid was performed in 2 mm cuvettes on the Bio-Rad Gene Pulser Electroporator (Model 165-2076). Following electroporation, 1 ml of glucose peptone yeast extract sucrose (GPYS) media (supplementary table S2, Supplementary Material online) was added and cells were incubated at 28 °C (no shaking) for 1 h and then plated on GPYS agar media with 100 µg/ml zeocin.

Visual screening for loss of pigmentation was used to identify putative Aurli_150841 knockout colonies, which were transferred and restreaked serially three times onto plates with zeocin-containing media, three times onto plates without zeocin in the media, and then again onto plates with zeocin-containing medium to confirm *shble* retention (data not shown).

#### Genomic DNA Extraction, PCR, and Southern Blotting

Genomic DNA was extracted according to a protocol that was based on Lippmeier et al. (2009) as outlined on protocols.io (https://dx.doi.org/10.17504/protocols.io.n83dhyn). LongAmp^®^*Taq* DNA Polymerase (NEB) was used with primers targeting the knockout region to identify *shble* integration into Aurli_150841 (150841_ORF_F and 150841_ORF_R; supplementary table S1, Supplementary Material online).

To perform a Southern blot, 2 μg of WT and putative knockout genomic DNA that were double digested with *Nde*I and *Hind*III were loaded on a 0.8% agarose gel and allowed to run for six hours at ∼40 V. Transfer, hybridization, wash, and detection were performed as indicated by the manufacturer (Roche) using a *shble* digoxigenin-labeled probe synthesized via PCR using PCR DIG Probe Synthesis Kit (Roche).

#### Nanopore Sequencing

##### DNA Extraction

WT *A. limacinum* ATCC MYA-1381 and putative knockout isolates 32 and 33 (referred to as KO32 and KO33, respectively) were cultured for three days in 50 ml 790 By + (supplementary table S2, Supplementary Material online). Genomic DNA was extracted as described above. The precipitated DNA was left to dissolve in water by spontaneous diffusion for 48+ hours at room temperature to avoid shearing and subsequently purified using QIAGEN Genomic-tip 20/G.

Agarose gel electrophoresis (1%) was used to visually assess and confirm the integrity of high molecular weight (20+ kbp) DNA. DNA quality was evaluated using a NanoPhotometer P360 (Implen) to measure A260/280 (∼1.8) and A260/230 (2.0–2.2) ratios. The quantity of DNA was calculated using a Qubit 2.0 Fluorometer (ThermoFisher Scientific) with the dsDNA broad range assay kit.

##### MinION Library Preparation and Sequencing

A multiplexed sequencing library for the WT and putative KOs was prepared using the Oxford Nanopore Technology (ONT) ligation sequencing kit (SQK-LSK109) and the PCR-free native barcoding expansion kit 1-12 (EXP-NBD103) according to the ONT protocol “1D Native barcoding genomic DNA with EXP-NBD103 and SQK-LSK109” (version NBE_9065_v109_revB_23May2018). The protocol modifications described below were made to optimize ligation steps and the retention of longer DNA fragments. Approximately 2 µg of purified genomic DNA per sample was used as input.

Unfragmented genomic DNA for the WT and putative KOs was repaired using the NEBNext formalin-fixed, paraffin-embedded (FFPE) DNA repair module (NEB cat. no. M6630) and prepared for adapter ligation using the NEBNext End repair/dA-tailing module (NEB cat. no. E7546) with incubations at 20 °C and 65 °C for 10 min each. The DNA repaired/end-prepped samples were purified with a 1:1 volume of AMPure XP beads (Beckman Coulter), and subjected to incubation at room temperature for 10 min; the pelleted beads were subsequently washed twice with 80% ethanol. The DNA was eluted off the beads in 25 µl nuclease free water for 10 min at 37 °C to encourage the elution of long molecules from the beads. The native barcodes NB07, NB08, and NB09 were ligated to the WT, KO32, and KO33 repaired/end-prepped DNA samples, respectively, using a 1-h incubation at room temperature. Each native barcoded sample was pooled in approximately equimolar amounts (∼1.3 µg each). The 1D barcode sequencing adapters (1D) were then ligated to the pooled and barcoded DNA in a 1.36× scaled ligation reaction and incubated for 1 h at 25 °C. The adapter-ligated DNA was purified by a 0.4× AMPure XP bead clean-up including a 10-min incubation at room temperature and two washes using the Long Fragment Buffer mix to enrich for DNA fragments >3 kbp. The final adapter-ligated library was incubated in 15 µl Elution Buffer for 10 min at 37 °C. A total of 1.2 µg of the prepared library was loaded on a single MinION R9.4.1 chemistry SpotON flow cell (FLO-MIN106) and sequenced via ONT's MinKNOW software (v2.1.12) without live basecalling. The raw fast5 MinION data has been deposited in the NCBI SRA database BioProject PRJNA680238 (WT accession: SRR13108467; KO32 accession: SRR13108466; KO33 accession: SRR13108465).

##### MinION Data Processing

Binning of the raw reads was performed in real time using Deepbinner v0.2.0 (https://github.com/rrwick/Deepbinner) and the demultiplexed fast5 files were subsequently base called using Albacore v2.3.1 (https://nanoporetech.com/). Only fastq sequences assigned to barcodes NB07, NB08, and NB09 were used for further analysis and the unsorted or miss-assigned files were disregarded. Adapters were removed by Porechop v0.2.3 (https://github.com/rrwick/Porechop). The resulting data were used for preliminary genome assembly by Canu v1.7.1 (https://github.com/marbl/canu) with parameters adjusted to the expected genome size of 60 Mbp. The resulting consensus sequence was improved by Nanopolish v0.10.1 (https://github.com/jts/nanopolish), resulting in finalized de novo genome assemblies for WT and both KO mutants. For WT, the genome assembly totaled 61.9 Mbp in 55 contigs. The genomes of KO mutants 32 and 33 both assembled as 62.5 Mbp into 50 and 47 contigs, respectively. The transgene insertion sites were localized to particular contigs in mutants 32 and 33 by BLAST (Altschul et al. 1990) using the *shble* gene as a query and its WT structure was determined using global alignment by Mauve (Darling et al. 2004) and local alignment by multiple alignment using fast fourier transform (MAFFT) (Katoh and Standley 2013).

Additionally, sequencing summary files produced by Albacore were used to assess sequencing data quality by Nanoplot v1.0.0 (https://github.com/wdecoster/NanoPlot). These summaries, as well as genome assembly details are available in supplementary table S3, Supplementary Material online.

#### Carotenoid Extraction and Quantification

From 235 h-cultures grown in GPY, 1.5 ml of cells (ranging in mass between 74 and 80 mg) were pelleted. To each tube, 250 mg of 0.5 mm glass beads and 1 ml of 100% acetone were added, vortexed for 30 min, and then centrifuged for 15 min at 4,000 rpm, at room temperature. The absorbance of the supernatant was measured by spectrophotometry (every half nanometer from 400–800 nm). Spectra were zeroed at 600 nm. The absorbance value at 454 nm, the extinction coefficient of β-carotene in acetone (134 × 10^3^ mo/lcm^−1^), and the molar mass of β-carotene (536.88 g/mol) were used in the conversion of absorbance to pigment mass (mg/g wet cell biomass).

#### Knockout and WT Growth Curves

Wild-type and knockout 32 were precultured in 5 ml GPY or 790 By + media with 100 µg/ml ampicillin, incubated at 28 °C, 170 rpm overnight, and subsequently inoculated into 45 ml GPY or 790 By + (50 ml total starting volume) with 100 µg/ml ampicillin. Optical density (OD600) was measured using an Infinite 200 PRO plate reader (Tecan) at 595 nm for 90 h by removing 500 µl of culture and loading triplicate wells with 150 µl each.

### Phylogenetics

#### Comparative Database Construction

A database of 36,866,870 predicted proteins representing 4,351 unique species from 117 phyla (supplementary table S4, Supplementary Material online) was constructed using the UniProt reference proteome at the 35% co-membership threshold including 4,295 representative proteome groups (Chen et al. 2011) in addition to all taxonomically identifiable transcriptomes of the Marine Microbial Eukaryote Transcriptome Sequencing Project (Keeling et al. 2014) that were processed through WinstonCleaner (https://github.com/kolecko007/WinstonCleaner). The database also included proteins inferred from the annotated and assembled genomes of *A. limacinum* ATCC MYA-1381, *S. aggregatum* ATCC 28209, and *Aplanochytrium kerguelensis* PBS07 from the U.S. Department of Energy's Joint Genome Institute, all PFAM PF00494 *Aurantiochytrium* sp. KH105 proteome hits from the Okinawa Institute of Science and Technology Marine Genomics Unit genome browser, all of UniProt’s annotated *H. fermentalgiana* proteins, and the annotated proteins of the breviate *Lenisia limosa* and associated mutualistic epibionts (Hamann et al. 2016).

#### Phylogenetic Analyses

The corresponding protein families of CrtIBY domains were identified by the National Center for Biotechnology Information (NCBI) Conserved Domain Database (CDD). The associated hidden Markov models (HMM) (supplementary table S5, Supplementary Material online) were used in conjunction with HMMER’s (3.3; hmmer.org/) hmmsearch to extract conserved domains from our custom comparative database. Resulting amino acid sequences were assigned and parsed according to orthologous groups by OrthoMCL (supplementary table S6, Supplementary Material online) (Chen et al. 2006) and R version 3.4.4 (R Core Team 2017).

Sequences longer or shorter than one standard deviation from the median length of the sequences within each orthologous group of interest were removed. Incomplete or split protein sequences (duplicate pairs) originally selected by hmmsearch were addressed by using fastafetch to retrieve the entire protein sequence and were subsequently aligned with hmmalign (−trim). Intermediate phylogenetic trees were made using FastTree version 2.1.9 SSE3 (Price et al. 2010) and dereplicated at a desired taxonomic level using a custom script (available on github.com: https://github.com/marianarius/carotenoidbiosynthesis/tree/master/Code/dereplication). The R script uses the original hmmsearch output and phytools 0.6–44 (Revell 2012) to identify and drop sisters with matching taxa, retaining the one with the higher bit score. The remaining sequences were again aligned (hmmalign) and a second intermediate phylogeny (FastTree) was made and again dereplicated (the second dereplication was omitted in *crtYc/d* and *blh* phylogenies due to the already relatively small number of sequences). Sequences from the second (or first for *crtYc/d* and *blh*) dereplication (provided in Supplementary Data file) were aligned using MAFFT (Katoh and Standley 2013) and positions where 99 or 90% of sequences contained a gap were removed (alternate alignments were evaluated: see Supplementary material). Final maximum likelihood (ML) trees were inferred using IQ-TREE v. 1.6.6 (Nguyen et al. 2015). The best fitting model (Yang 1995; Soubrier et al. 2012) was selected following the Akaike information criterion and the Bayesian information criterion for each phylogeny (supplementary table S5, Supplementary Material online). The SH-aLRT and ultrafast bootstrap were calculated from 1,000 replicates. Phylogenies were midpoint rooted using phangorn (Schliep 2010) and visualized using ggtree (Yu et al. 2017) in R (version 4.1.1) (R Core Team 2017).

## Supplementary Material

evad029_Supplementary_Data

## Data Availability

The database used in the phylogenetic analyses is available at Dryad: https://doi.org/10.5061/dryad.4tmpg4ffn. The output from HMM, OrthoMCL, and dereplication are available with the alignments, trees, and code on https://github.com/marianarius/carotenoidbiosynthesis/tree/master. The resulting protein sequence accession IDs and their corresponding taxonomic classifications are provided for each investigated carotenogenic protein in the Supplementary material (.xlsx).
