# Supplementary material for: Horizontal Gene Transfer and Fusion Spread Carotenogenesis Among Diverse Heterotrophic Protists
Source: Genome Biol Evol. 2023 Feb 20;15(3):evad029. doi: 10.1093/gbe/evad029 (PMC10016063; doi:10.1093/gbe/evad029)
Supplement: evad029_Supplementary_Data [file evad029_supplementary_data.zip › Rius_SupplementaryMaterial_Revised_edit_Merged.pdf]

Supplementary Material

Horizontal Gene Transfer and Fusion Spread Carotenogenesis Among Diverse Heterotrophic Protists

Mariana Rius[1], Joshua S. Rest[2], Gina V. Filloramo[3], Anna M. G. Novák Vanclová[4], John M. Archibald[3], and Jackie L. Collier[1*]

1. School of Marine and Atmospheric Sciences, Stony Brook University, Stony Brook, NY, 11794 USA
2. Department of Ecology and Evolution, Stony Brook University, Stony Brook, NY, 11794-5245 USA
3. Department of Biochemistry and Molecular Biology, Dalhousie University, Halifax, Nova Scotia, Canada B3H4R2
4. Faculty of Science, Charles University, BIOCEV, Vestec 252 50, Czechia (current affiliation: Institut de Biologie de l'École Normale Supérieure, Paris 75005, France)

* Corresponding author
E-mail: jackie.collier@stonybrook.edu (JLC)

Supplementary Material

MAFFT and hmmalign alignment and tree comparisons

For each domain, six maximum-likelihood trees were evaluated. The six trees were made using alignments generated by either MAFFT (Katoh and Standley 2013) or hmmalign (hmmer.org) where either all sequence alignment positions were retained, positions where 99% of sequences were gaps were removed, or positions where 90% of sequences were gaps were removed (**Table S5**). All six maximum-likelihood trees for each domain were inferred using IQ-TREE v. 1.6.6 (Nguyen et al. 2015) with the same best-fitting (Yang 1995, Soubrier et al. 2012) model, selected following the Akaike information criterion and the Bayesian information criterion for each phylogeny (**Table S5**). All phylogenies maintained conserved lineage grouping, that is, for instance, CrtB and CrtI maintained the four lineages (Proteobacterial, photosynthetic eukaryotes, firmicutes, and ABA: Actinobacteria, bacteroidetes, and archaea). CrtYc/d, Blh, RPE65, and CrtY phylogenies maintained branch structure and did not exhibit within lineage rearrangements. The MAFFT alignments retaining positions where less than 90-99% of sequences containing gaps were selected for the reduced branch lengths.

H. fermentalgiana genomic annotation of A0A2R5GF32

The carotenoid biosynthesis gene in *H. fermentalgiana*: A0A2R5GF32, annotated as 'L-amino-acid oxidase', received a 1 out 5 annotation score. The gene (FCC1311_095101; version GBG29536) is described as containing two coding exons (*crtI, crtB*), 2,511 bps in length and 836 residues long. It is located on SuperContig BEYU01000059: 213,058 - 215,632. In exporting the BEYU01000059 (210,870 - 215,706) DNA supercontig, an open reading frame spanning 211,827 - 214,749 (in the reverse direction) appears when scanning for ORFs. An NCBI CDD BLASTp of this fragment reveals a trifunctional fused gene (*crtI, crtB, crtYc/d*) that is 1268 residues in length (3,858 bps) (**Figure 3B**).

BUSCO

BUSCO analyses were conducted on the single transcriptome available for *N. scintillans* and a concatenated transcriptome of *O. marina*, including all nine available MMETSP transcriptomes, to discern whether the undetectability of the heterodimeric lycopene cyclase (*crtYc/ crtYd)* gene was due to incompleteness of the *O. marina* transcriptomes. BUSCO analyses (BUSCO 3.0.1; Python 3.5.2) reveal that in *N. scintillans* and in *O. marina* 18.8% and 20.5%, respectively, is missing when evaluated using general eukaryotes (eukaryota_odb9; Creation date: 2016-11-02, number of species: 100, number of BUSCOs: 303).

References

Katoh K, Standley DM. 2013. MAFFT multiple sequence alignment software version 7: improvements in performance and usability. Mol. Biol. Evol. 30(4):772–780.

Oberto J. 2013. SyntTax: a web server linking synteny to prokaryotic taxonomy. BMC Bioinform. 14:4.

Soubrier J, Steel M, Lee MSY, Der Sarkissian C, Guindon S, Ho SYW, Cooper A. 2012. The Influence of Rate Heterogeneity among Sites on the Time Dependence of Molecular Rates. Mol Biol Evol. 29(11):3345–3358.

Yang Z. 1995. A space-time process model for the evolution of DNA sequences. Genetics. 139(2):993-1005.

Supplementary Tables

Table S1. Primer names, sequences, and amplification region used in the In-fusion cloning of Aurli_150841_GZG (Addgene Plasmid 162563).

Name	**Sequence (5' to 3')**	**Used in Amplification of**
**Acc65I LA_F**	ATTCGAGCTCGGTACCGCCACCCAGGATGTCTGTC	Aurli1_150841 Left arm with Acc65I
**Acc65I LA_R**	CAAGATCAAGGGTACCGGCTTAACCCTCCACTAGGG	Aurli1_150841 Left arm with Acc65I
**SphI RA_F**	TCTAGTGCTTCAAGGCATGCGATTTTTCCGCCGAGCTG	Aurli1_150841 Right arm with SphI
**SphI RA_R**	ATTACGCCAAGCTTGCATGCGCTCCAGAGTTCTCGAGTGC	Aurli1_150841 Right arm with SphI
**150841_ORF_F**	GTGTGAGAAGGTCTGCGACA	Aurli1_150841 Knockout region
**150841_ORF_R**	TGTCCCAAGGGGTGGTATAA	Aurli1_150841 Knockout region

Table S2. Composition of 790 By+, GPY, and GPYS media.

**Nutrient**	**790 By+ (%)**	**GPY (%)**	**GPYS (%)**
Glucose	0.5	3.0	3.0
Peptone	0.1	1.0	0.6
Yeast Extract	0.1	0.5	0.2
Instant Ocean	1.8	1.8	1.8
Sucrose	--	--	1.7

Table S3. NanoPlot report for WT, 32, 33.

**Summary statistics prior to filtering**	**WT**	**KO 32**	**KO 33**
Active channels:	503	503	504
Mean read length:	4913.5	8508.3	7951.5
Mean read quality:	9.3	9.3	9.3
Median read length:	2303	3877	3499
Median read quality:	9.6	9.6	9.6
Number of reads:	345135	258971	238315
Read length N50:	11327	20120	19624
Total bases:	1695835482	2203401663	1894970894
**Reads and Megabases above quality cutoffs**	**WT**	**KO 32**	**KO 33**
>Q5:	342752 (99.3%) 1689.0Mb	257360 (99.4%) 2196.1Mb	236823 (99.4%) 1889.0Mb
>Q7:	322874 (93.6%) 1618.5Mb	242777 (93.7%) 2117.8Mb	223434 (93.8%) 1822.2Mb
>Q10:	120657 (35.0%) 636.6Mb	90157 (34.8%) 829.9Mb	82854 (34.8%) 713.9Mb
>Q12:	567 (0.2%) 0.3Mb	898 (0.3%) 0.3Mb	643 (0.3%) 0.2Mb
>Q15:	0 (0.0%) 0.0Mb	0 (0.0%) 0.0Mb	0 (0.0%) 0.0Mb
**Top 5 quality scores and read lengths**	**WT**	**KO 32**	**KO 33**
1:00	14.9 (267)	14.6 (245)	14.4 (184)
2:00	14.7 (191)	14.6 (267)	14.4 (249)
3:00	14.1 (237)	14.5 (154)	14.1 (308)
4:00	14.0 (129)	14.4 (210)	14.0 (170)
5:00	13.6 (138)	14.4 (179)	13.9 (216)
**Top 5 longest reads and quality score**	**WT**	**KO 32**	**KO 33**
1:00	122423 (10.3)	180475 (9.8)	130269 (9.7)
2:00	121698 (8.8)	153580 (7.4)	126830 (8.6)
3:00	111237 (7.8)	138783 (7.8)	122652 (8.7)
4:00	104456 (10.6)	131259 (10.8)	116043 (9.0)
5:00	103754 (7.9)	125687 (8.0)	116037 (7.5)

Table S4. Unique phyla represented in the reference proteome database.

Archaea	Bacteria		Eukaryota	
Candidatus	Abditibacteriota	Firmicutes	Annelida	Fornicata
Caudovirales	Acidobacteria	Fusobacteria	Apicomplexa	Glaucocystophyceae
Crenarchaeota	Actinobacteria	Gemmatimonadetes	Apusomonadidae	Haptista
Euryarchaeota	Aquificae	Haloplasmatales	Arthropoda	Heterolobosea
Hyperthermophilic	Armatimonadetes	Ignavibacteriae	Ascomycota	Ichthyosporea
Nanoarchaeota	bacterium	Kiritimatiellaeota	Bacillariophyta	Microsporidia
Thaumarchaeota	Bacteroidetes	Lentisphaerae	Basidiomycota	Mollusca
	Balneolaeota	Natronospirillum	Bigyra	Mucoromycota
	Calditrichaeota	Nitrospinae	Blastocladiomycota	Nematoda
	candidate	Nitrospirae	Bolidophyceae	Oomycetes
	Candidatus	Planctomycetes	Brachiopoda	Palpitomonas
	Chlamydiae	Proteobacteria	Breviatea	Parabasalia
	Chlorobi	Rhodothermaeota	Cercozoa	Pelagophyceae
	Chloroflexi	Spirochaetes	Chlorophyta	Perkinsozoa
	Chrysiogenetes	Synergistetes	Choanoflagellata	Phaeophyceae
	Coprothermobacterota	Tenericutes	Chordata	Pinguiophyceae
	Cyanobacteria	Thermobaculum	Chromeraceae	Placozoa
	Deferribacteres	Thermodesulfobacteria	Chromerida	Platyhelminthes
	Deinococcus-Thermus	Thermotogae	Chrysophyceae	Porifera
	Dictyoglomi	Vampirococcus	Chytridiomycota	Raphidophyceae
	Elusimicrobia	Verrucomicrobia	Ciliophora	Rhodophyta
	Fibrobacteres		Cnidaria	Rotifera
			Cryptomycota	Rotosphaerida
			Cryptophyta	Stereomyxa
			Dictyochophyceae	Streptophyta
			Dinophyceae	Synchromophyceae
			Discosea	Synurophyceae
			Echinodermata	Tardigrada
			Endomyxa	Tubulinea
			Euglenozoa	Vitrellaceae
			Evosea	Xanthophyceae
			Filasterea	Zoopagomycota
			Foraminifera	

Table S5. Pipeline progression in number of sequences and alignment lengths.

	CrtB	CrtI	CrtYc/d	CrtY	Blh	Rpe65
**HMM ACC**	PF00494.19	TIGR02734	TIGR03462	PF05834.12	PF15461.6	PF03055.15
**Description**	Squalene/ phytoene synthase	crtI_fam: phytoene desaturase	CarR_dom_SF: lycopene cyclase domain	Lycopene cyclase protein	Beta-carotene 15,15'-dioxygenase	Retinal pigment epithelial membrane protein
**HMM length**	263 aa	504 aa	89 aa/ 227 aa	380 aa	268 aa	463 aa
**hmmsearch**	6616 seq	32241 seq	697 seq	5408 seq	199 seq	6454 seq
**OrthoMCL return total**	6369 seq	21902 seq	NA	3639 seq	NA	6186 seq
**OrthoMCL**	OG5_131363	OG5_135453	NA	OG5_143063	NA	OG5_128633
**OrthoMCL OG5 name**	phytoene synthase	phytoene dehydrogenase	NA	Lycopene cyclase protein	NA	Retinal pigment epithelial membrane protein
**OG5 # seqs**	3466 seq	2204 seq	NA	922 seq	NA	2123 seq
**Seq size filter result**	3054 seq	1716 seq	417 seq	748 seq	177 seq	1701 seq
**hmmalign x1 length**	878 aa	2250 aa	550 aa	1498 aa	488 aa	2073 aa
**derep. x1 remaining**	1335 seq	859 seq	238 seq	268 seq	95 seq	745 seq
**hmmalign x2 length**	647 aa	1945 aa	NA	1352 aa	NA	1803 aa
**derep x2 remaining**	1247 seq	812 seq	NA	260 seq	NA	734 seq
**final hmmal length**	647 aa	1705 aa	550 aa	1333 aa	463 aa	1803 aa
**final hmmal - 99% gap**	361 aa	697 aa	305 aa	887 aa	NA	988 aa
**final hmmal - 90% gap**	281 aa	528 aa	251 aa	469 aa	294 aa	636 aa
**MAFFT length**	1070 aa	1631 aa	617 aa	1264 aa	652 aa	3226 aa
**MAFFT length - 99% gap**	480 aa	890 aa	389 aa	778 aa	NA	1457 aa
**MAFFT length - 90% gap**	304 aa	538 aa	294 aa	519 aa	358 aa	690 aa
**IQ-TREE model**	WAG+F+R10	LG+F+R10	VT+F+R6	WAG+F+R9	LG+F+R7	VT+F+R10

Table S6. Orthologous group (OG) classification and distribution across HMMs by OrthoMCL. Bold indicates OG selected for phylogenetic analysis.

HMM	OGs	Name	Sequences	%
PF00494	OG5 129470	squalene synthase	1485	26
	**OG5 131363**	**phytoene synthase**	**3020**	**53**
	OG5 130108	hypothetical protein	1197	21
TIGR02734	OG5 126892	Conserved region in glutamate synthase	3104	10.618137
	**OG5 135453**	**phytoene dehydrogenase**	**2180**	**7.457326**
	OG5 131173	n/a	2005	6.858687
	OG5_133149	n/a	1643	5.620361
	NO_GROUP	n/a	1540	5.268019
	OG5_144826	n/a	1475	5.045668
	OG5_136205	Flavin containing amine oxidoreductase	1265	4.327301
	OG5_129066	Flavin containing amine oxidoreductase	1038	3.550782
	OG5_130448	Flavin containing amine oxidoreductase	917	3.136866
	Other OGs	--	14088	48
TIGR0346	**220 OGs**	**--**	**891**	**100**
PF05834	**OG5 143063**	**Lycopene cyclase protein**	**1023**	**28**
	OG5_130321	FAD-binding domain	616	17
	124 Other OGs	--	2000	55
PF15461	OG5_233550	n/a	44	75
	OG5_233492	n/a	15	25
PF03055	OG5 131329	Retinal pigment epithelial membrane protein	2709	44
	**OG5 128633**	**Retinal pigment epithelial membrane protein**	**2205**	**36**
	9 Other OGs	--	1272	21

Supplementary Figure Captions and Figures

Fig. S1. *crtIBY* inactivation plasmid (Aurli_150841_GZG Plasmid: addgene.org/162563; sequence diagrams generated using SnapGene). (**A**) Map of Aurli_150841 (*crtIBY*) open reading frame (ORF) and conserved domains: phytoene desaturase (*crtI*), phytoene synthase (*crtB*), and heterodimeric lycopene cyclase domains (lyc…) (*crtYc/d*). The 2 kb arms of homology (Aurli_150841_Left_Arm and Aurli_150841_Right_Arm) are indicated. (**B**) Plasmid map of Aurli_150841_GZG. Native GAPDH promoter/ terminator system drives *shble/* BleoR expression. Restriction enzyme AvrII was used to linearize the fragment of interest.

Fig. S2. Pigment and growth of wild-type (WT) and *crtIBY* knockout (32). (**A**) Absorbance spectra of extracted pigments from *crtIBY* knockout (32) and WT cultured in GPY media for 7 days. Carotenoid accumulation is calculated using the absorbance peak at 454 nm. In the *crtIBY* knockout (32), carotenoid accumulation is below the level of detection, while in WT carotenoid accumulation is 9.4 mg/g wet weight. (**B**) Mean optical density of WT and 32 grown in 50 ml cultures of GPY or 790 media over 89 hours. Error bars represent standard deviation of technical triplicates.

Fig. S3. PCR and Southern blot verification of inactivation plasmid integration in *crtIBY* locus in the two knockouts: 32 and 33. Annotated DNA sequences were generated using SnapGene. (**A**) Diagram of 3.6 kb knockout (KO) *crtIBY* open reading frame (ORF) amplification product with 150841_ORF primers. (**B**) Diagram of 2.9 kb Wild-type (WT) *crtIBY* ORF amplification product with 150841_ORF primers. (**C**) Gel electrophoresis of 150841_ORF PCR products. KOs 32 and 33, and plasmid (pla) amplified ~3.6 kb product. WT (non-transformed *A. limacinum*) amplified ~2.6 kb PCR product. (**D**) Diagram of KOs 4.8 kb Southern blot product resulting from NdeI and HindIII digested genomic DNA when hybridized using a *shble* probe. (**E**) Southern blot of 32 and 33 (on the left) was developed using a DIG-labeled *shble* probe and NdeI and HindIII. Digested genomic DNA was run on gel electrophoresis (on the right).

Fig. S4. Maximum likelihood tree of CrtB/ CrtM/ HpnD domains aligned with MAFFT and retaining positions where less than 99% of sequences were gaps. Values indicate SH-aLRT and ultrafast bootstrap from 1000 replicates in IQ-TREE using model LG+F+R10. SAHNTO represents a cluster of thraustochytrids: *A. limacinum, S. aggregatum, H. fermentalgiana*, two dinoflagellates: *N.*

scintillans, O. marina, and an apusomonad: *T. trahens*. Node points indicate SH-aLRT support > 80% and/ or ultrafast bootstrap support >95%.

Fig. S5. Maximum likelihood tree of CrtI/ CrtH/ Z-ISO/ CRISTO domains aligned with MAFFT and retaining positions where less than 90% of sequences were gaps. Values indicate SH-aLRT and ultrafast bootstrap from 1000 replicates in IQ-TREE using model WAG+F+R10. SAHNTO represents a cluster of thraustochytrids*: A. limacinum, S. aggregatum, H. fermentalgiana*, two dinoflagellates: *N. scintillans, O. marina*, and an apusomonad: *T. trahens.* Node points indicate SH-aLRT support > 80% and/ or ultrafast bootstrap support >95%.

Fig. S6. Maximum likelihood tree of CrtYc/d domains aligned with MAFFT and retaining positions where less than 90% of sequences were gaps. Values indicate SH-aLRT and ultrafast bootstrap from 1000 replicates in IQ-TREE using model VT+F+R6. SAHNTO represents a cluster of thraustochytrids: *A. limacinum, S. aggregatum*, two sequences from the dinoflagellate: *N. scintillans,* and an apusomonad: *T. trahens.* Node points indicate SH-aLRT support > 80% and/ or ultrafast bootstrap support >95%.

Fig. S7. Maximum likelihood tree of CrtY/ CrtL/ LCY-b domains aligned with MAFFT and retaining positions where less than 90% of sequences were gaps. Values indicate SH-aLRT and ultrafast bootstrap from 1000 replicates in IQ-TREE using model WAG+F+R9. Node points indicate SH-aLRT support > 80% and/ or ultrafast bootstrap support >95%.

Fig. S8. Maximum likelihood tree of Blh domains aligned with MAFFT and retaining positions where less than 90% of sequences were gaps. Values indicate SH-aLRT and ultrafast bootstrap from 1000 replicates in IQ-TREE using model LG+F+R7. SAHNTO represents a cluster of a dinoflagellate: *O. marina* and an apusomonad: *T. trahens*. Node points indicate SH-aLRT support > 80% and/ or ultrafast bootstrap support >95%.

Fig. S9. Maximum likelihood tree of Rpe65 domains aligned with MAFFT and retaining positions where less than 90% of sequences were gaps. Values indicate SH-aLRT and ultrafast bootstrap from 1000 replicates in IQ-TREE using model VT+F+R10. Node points indicate SH-aLRT support > 80% and/ or ultrafast bootstrap support >95%.

Fig. S10. Diagram of *Mycobacterium* spp*.* operon reveals *crtI* (tan arrow)*, crtB* (blue arrow)*, crtYc* (small white arrow)*, crtYd* (small white arrow) gene organization (SyntTax; Oberto 2013).

Fig. S11. Diagram of *Mycolicibacterium* spp*.* operon reveals *crtI* (tan arrow)*, crtB* (blue arrow)*, crtYc* (small white arrow)*, crtYd* (small white arrow) gene organization (SyntTax; Oberto 2013).

Fig. S12. Diagram of *Nocardia* spp*.* operon reveals *crtI* (blue arrow)*, crtB* (pink arrow)*, crtYc* (small white arrow)*, crtYd* (small white arrow) gene organization (SyntTax; Oberto 2013).

Fig. S13. Diagram of *Haloarcula* spp*.* operon reveals *crtB* (yellow arrow)*, crtYc/d* (green arrow)*, blh* (blue arrow) gene organization (SyntTax; Oberto 2013).

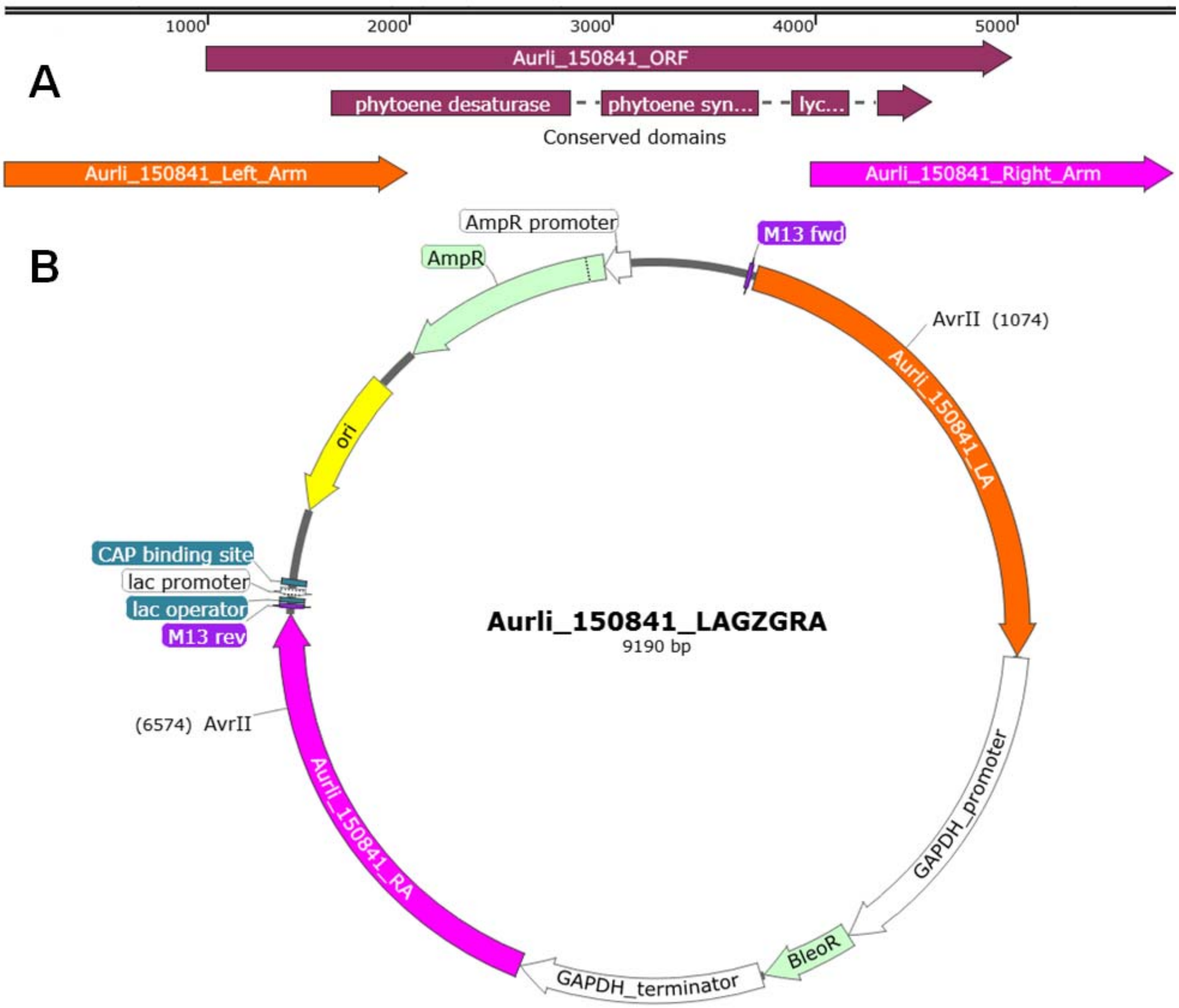

Fig. S1. Rius et al.

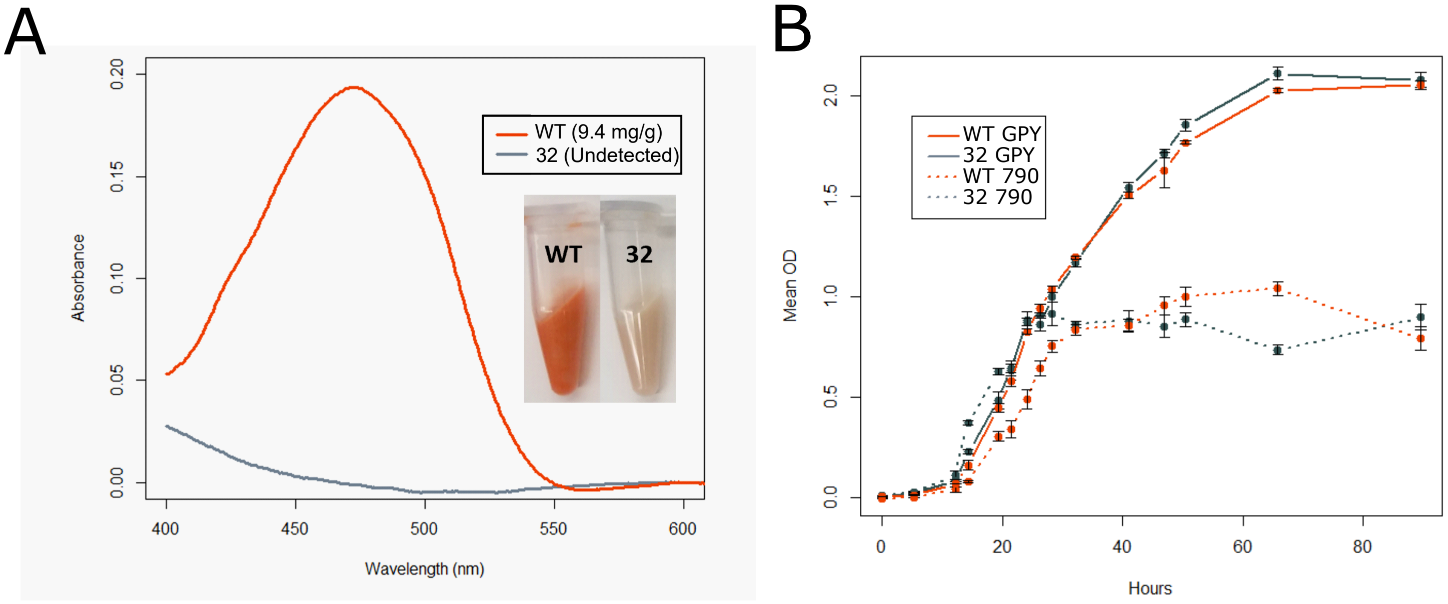

Fig. S2. Rius, et al.

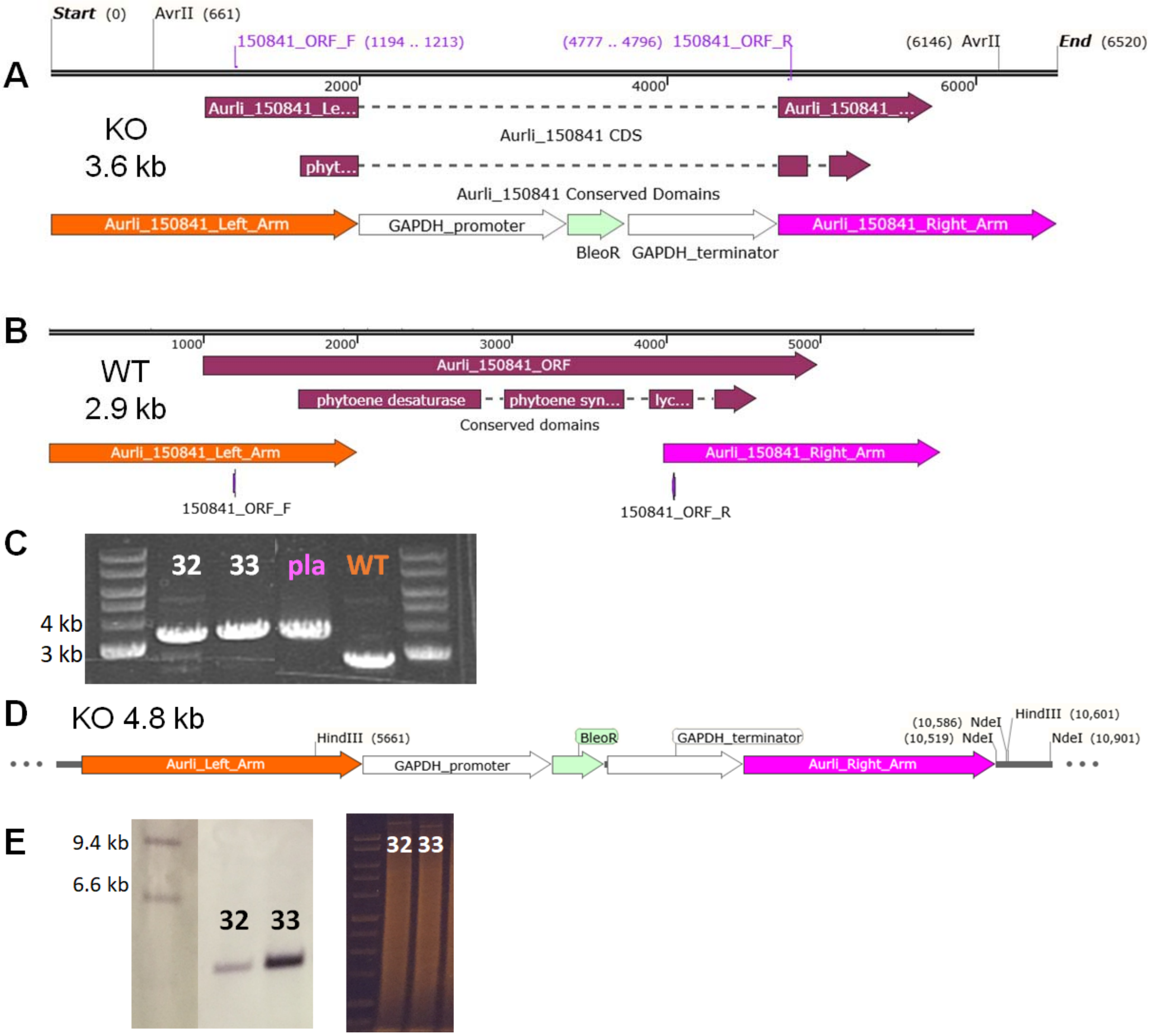

Fig. S3. Rius et al.

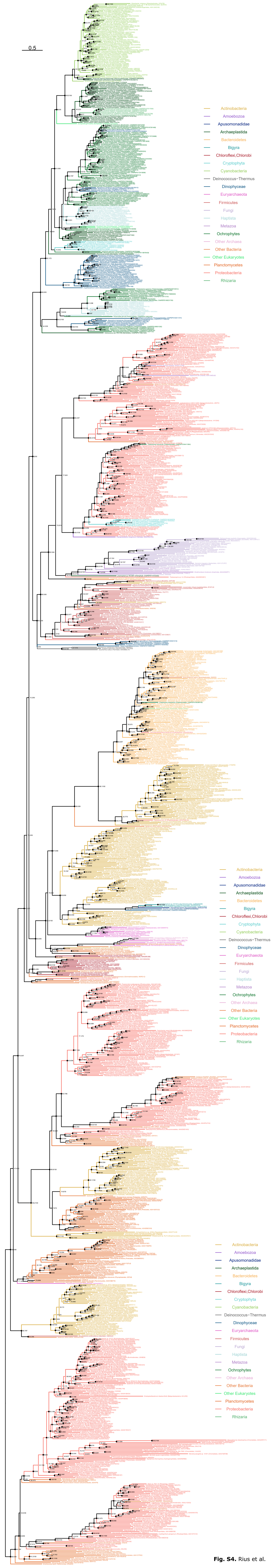

Fig. S4. Rius et al.

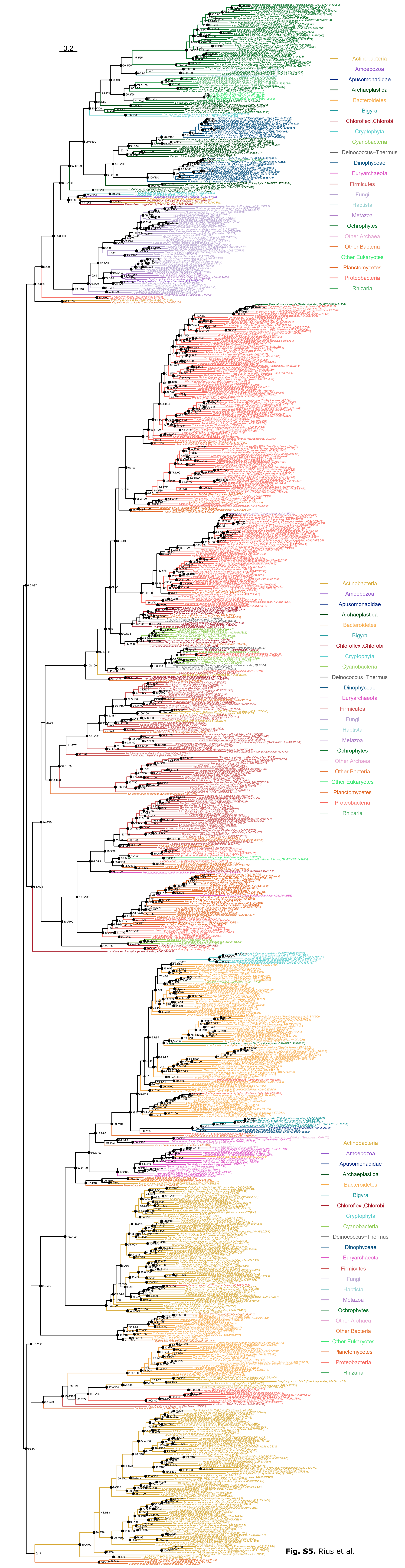

Fig. S5. Rius et al.

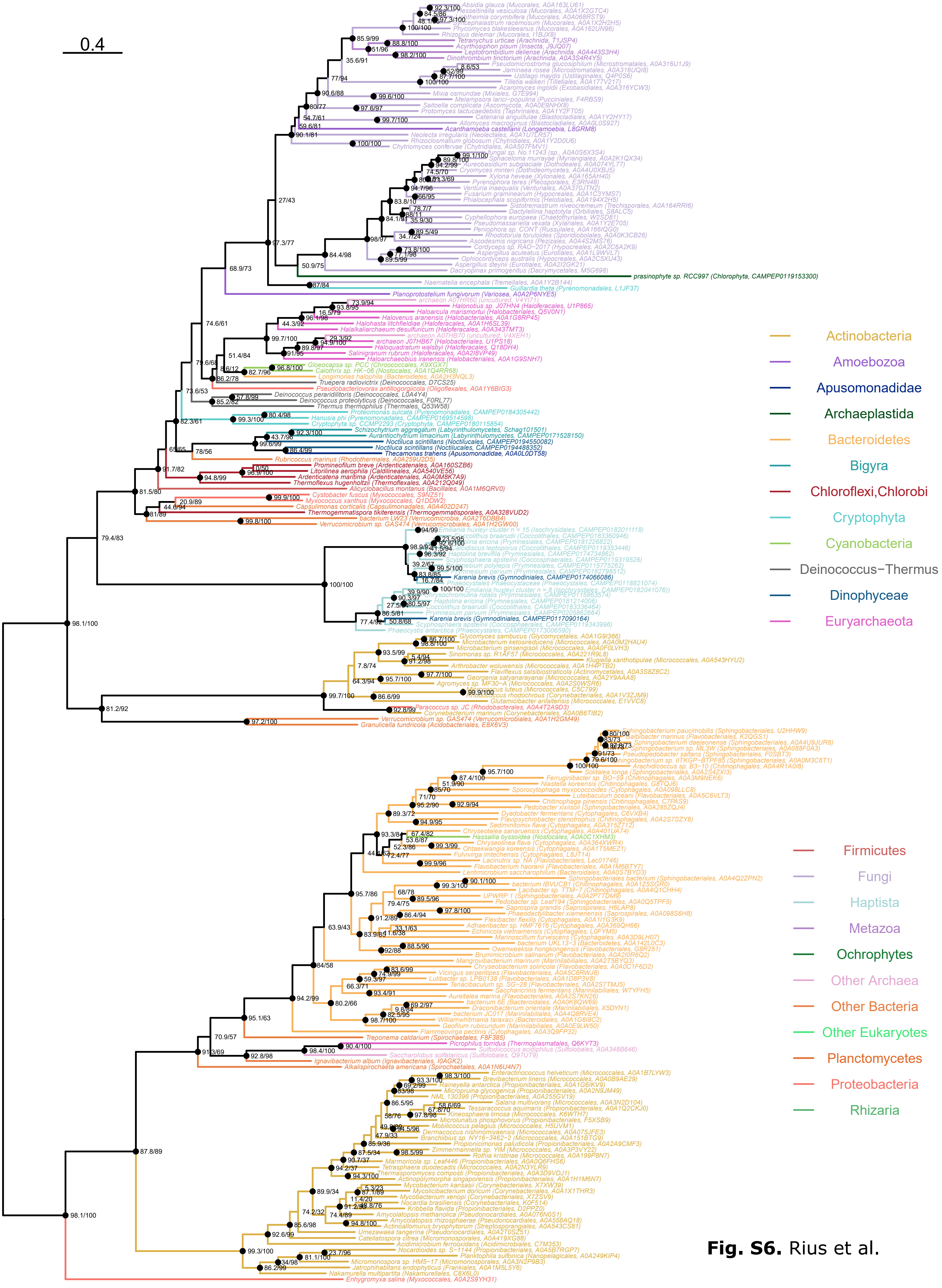

Fig. S6. Rius et al.

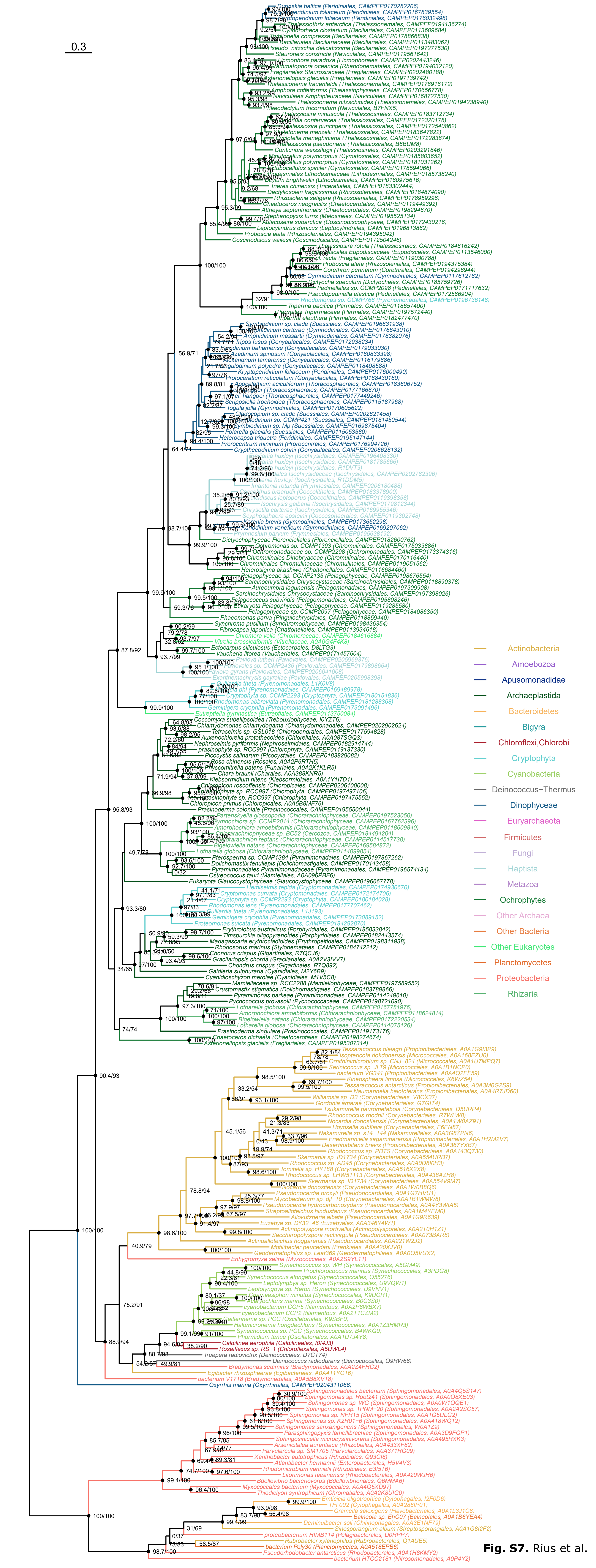

Fig. S7. Rius et al.

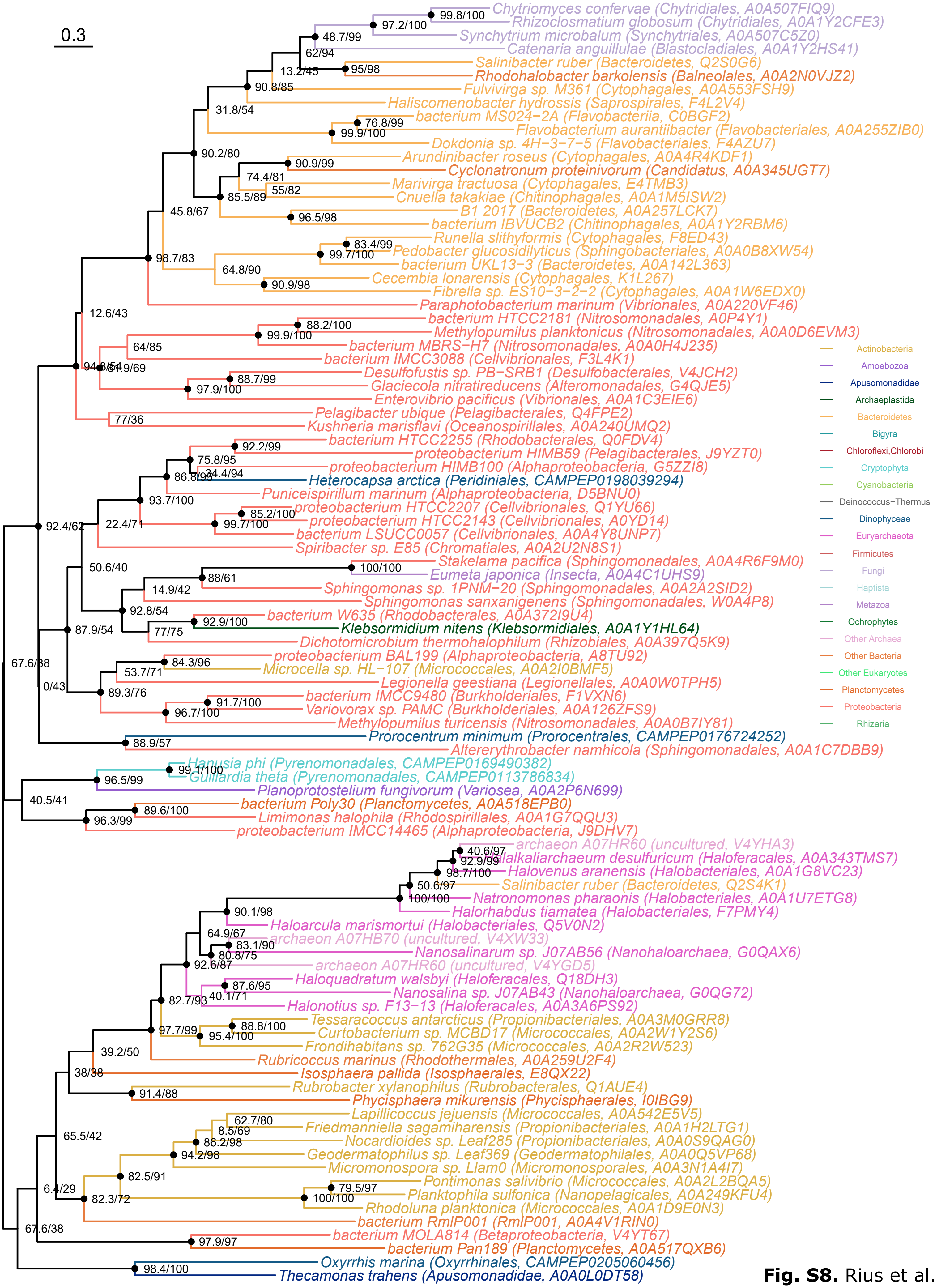

Fig. S8. Rius et al.

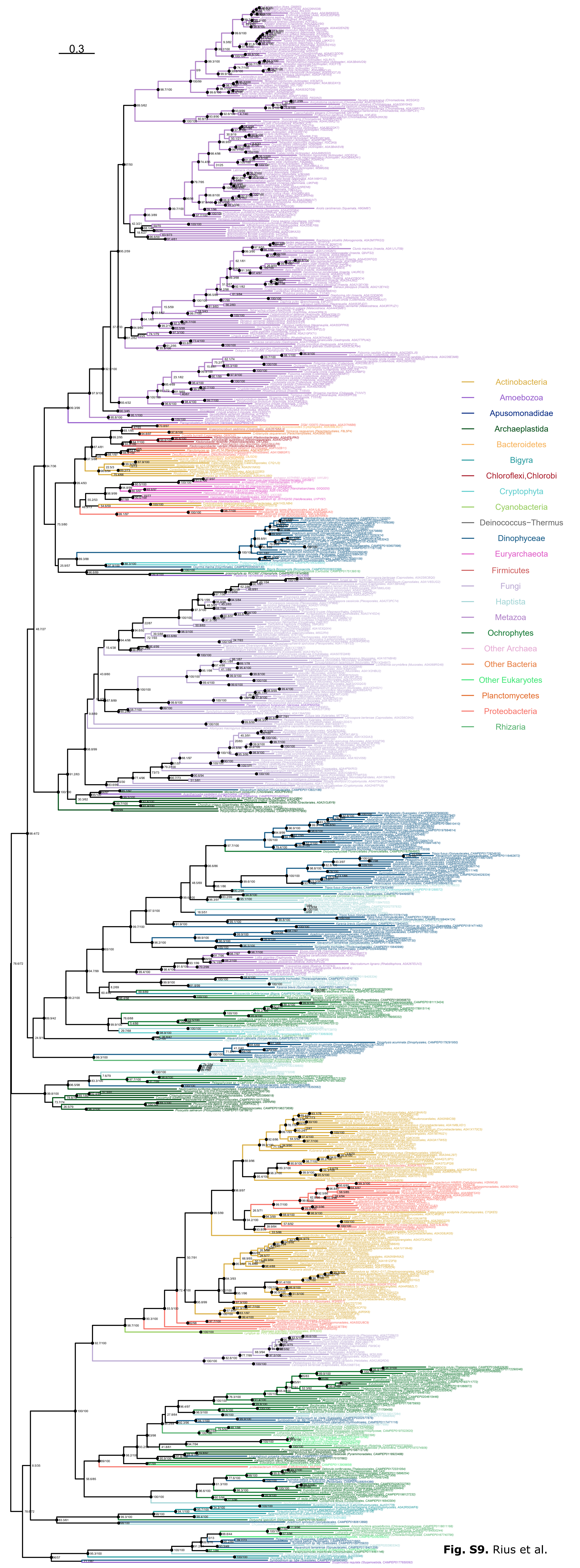

Fig. S9. Rius et al.

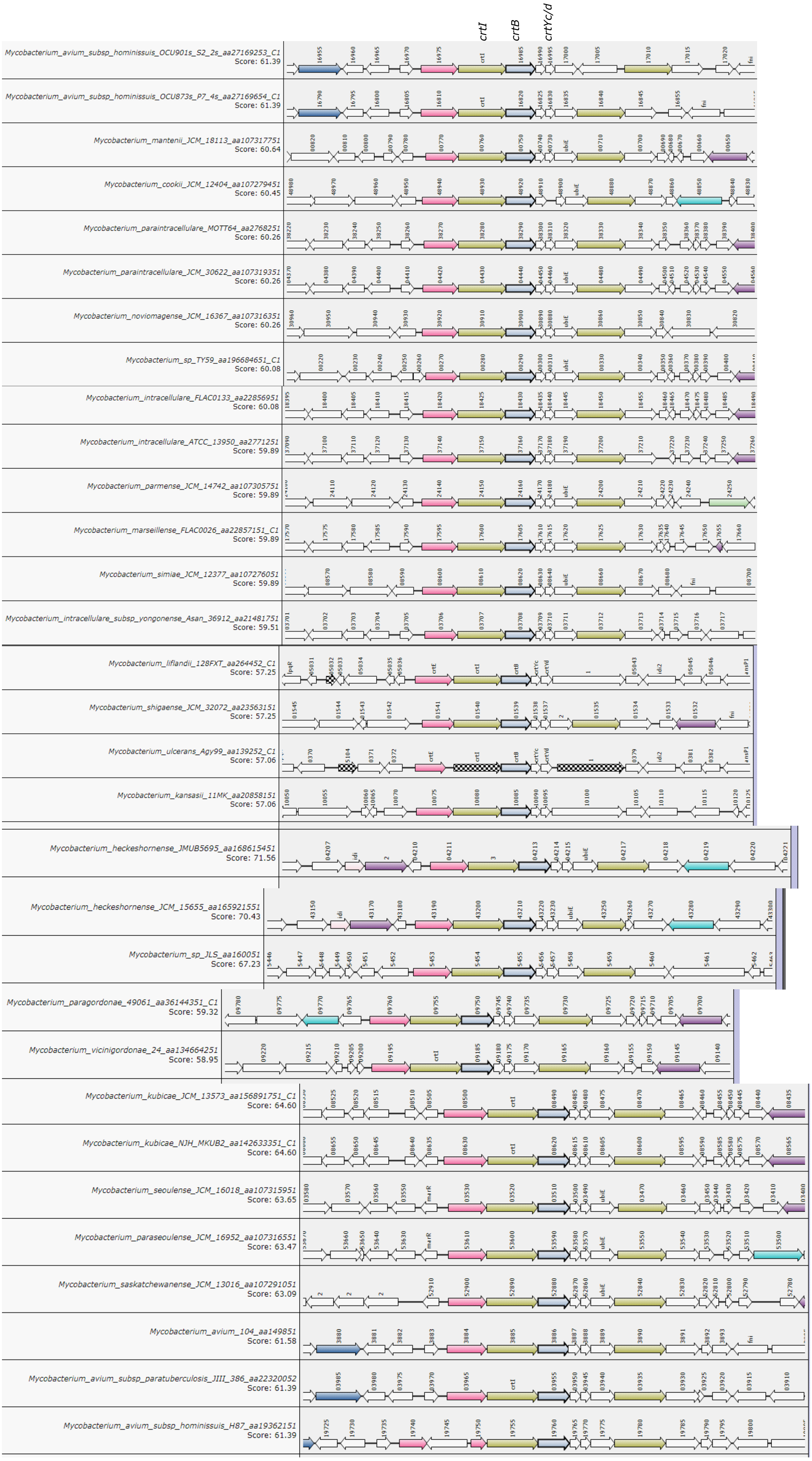

Fig. S10.
Rius et al.

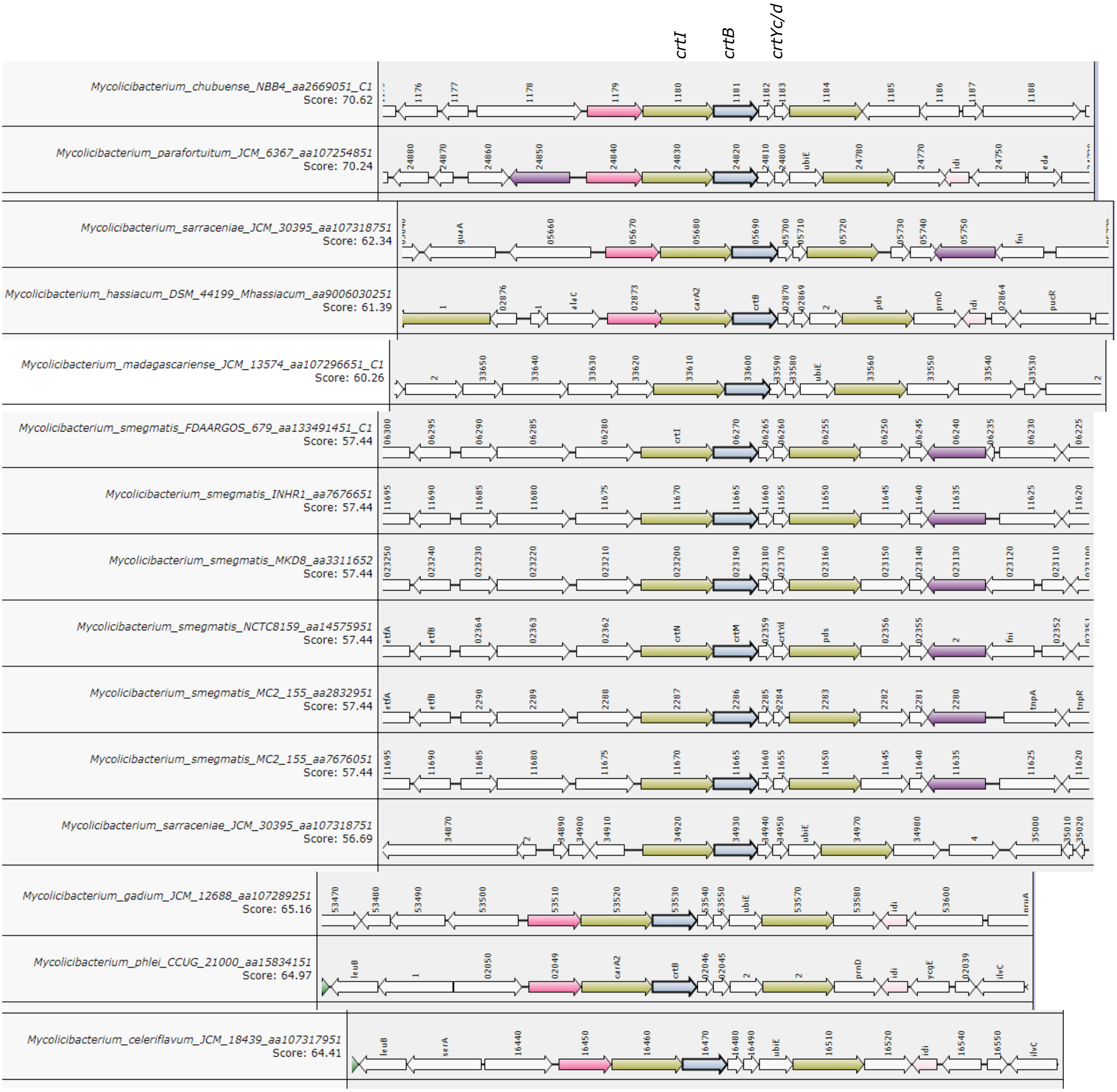

Fig. S11. Rius et al.

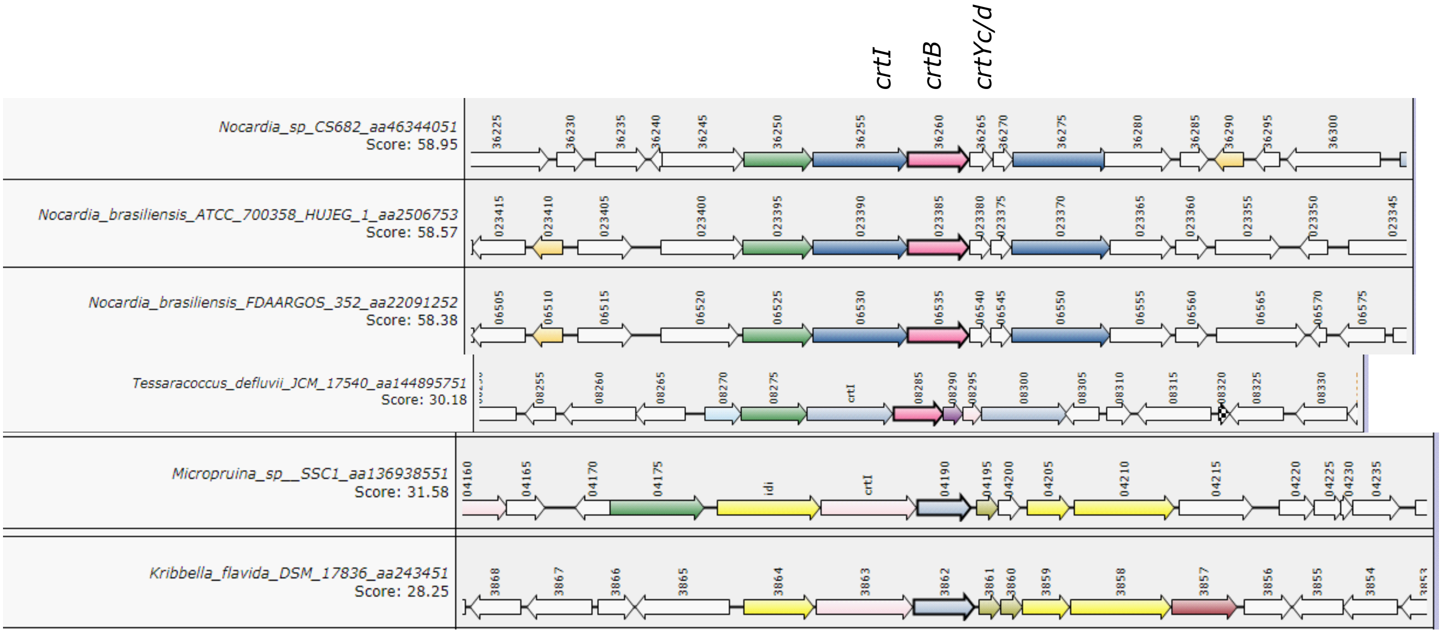

Fig. S12. Rius et al.

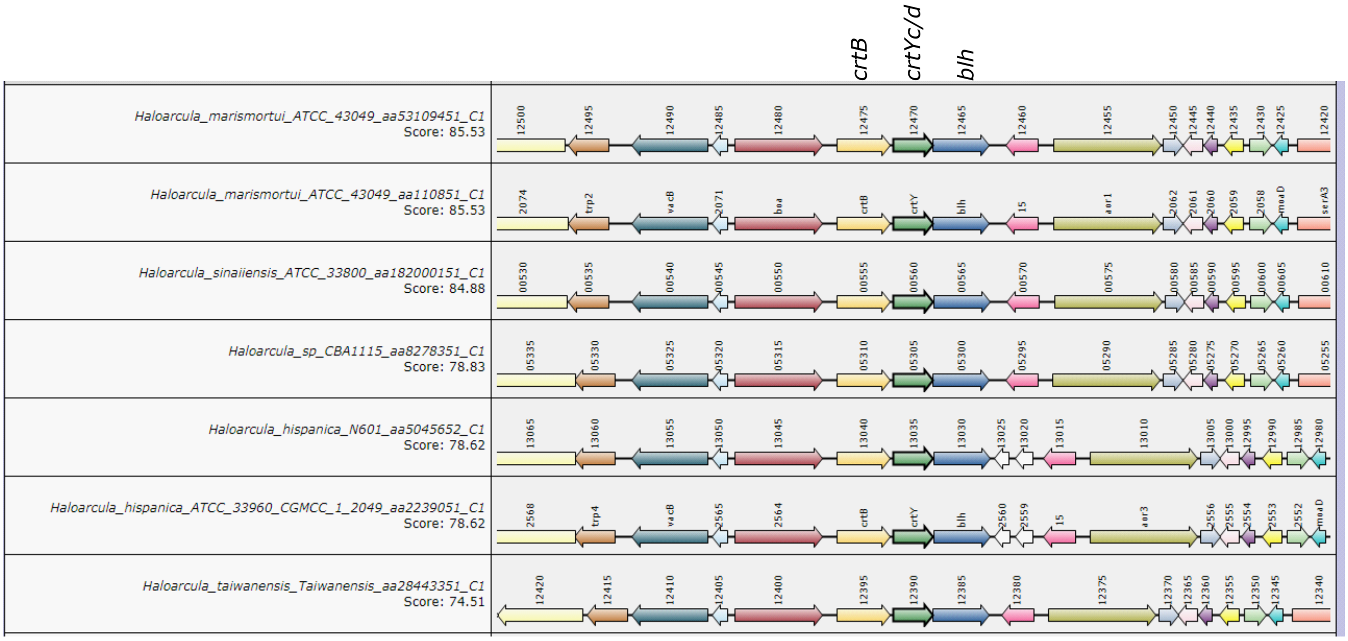

Fig. S13. Rius et al.
